# Neoplastic ICAM-1 protects lung carcinoma from apoptosis through ligation of fibrinogen

**DOI:** 10.1038/s41419-024-06989-9

**Published:** 2024-08-21

**Authors:** ShiHui Wang, JunLei Wang, Cui Liu, Lei Yang, XuanQian Tan, ShiYang Chen, Yun Xue, HongBin Ji, GaoXiang Ge, JianFeng Chen

**Affiliations:** 1 State Key Laboratory of Multi-Cell Systems, Shanghai Institute of Biochemistry and Cell Biology, Center for Excellence in Molecular Cell Science, Chinese Academy of Sciences, Shanghai, China; 2 Key Laboratory of Systems Health Science of Zhejiang Province, School of Life Science, Hangzhou Institute for Advanced Study, University of Chinese Academy of Sciences, Hangzhou, China

**Keywords:** Non-small-cell lung cancer, Apoptosis

## Abstract

Intercellular cell adhesion molecule-1 (ICAM-1) is frequently overexpressed in non-small cell lung cancer (NSCLC) and associated with poor prognosis. However, the mechanism underlying the negative effects of neoplastic ICAM-1 remains obscure. Herein, we demonstrate that the survival of NSCLC cells but not normal human bronchial epithelial cells requires an anti-apoptosis signal triggered by fibrinogen γ chain (FGG)–ICAM-1 interaction. ICAM-1–FGG ligation preserves the tyrosine phosphorylation of ICAM-1 cytoplasmic domain and its association with SHP-2, and subsequently promotes Akt and ERK1/2 activation but suppresses JNK and p38 activation. Abolishing ICAM-1–FGG interaction induces NSCLC cell death by activating caspase-9/3 and significantly inhibits tumor development in a mouse xenograft model. Finally, we developed a monoclonal antibody against ICAM-1–FGG binding motif, which blocks ICAM-1‒FGG interaction and effectively suppresses NSCLC cell survival in vitro and tumor growth in vivo. Thus, suppressing ICAM-1–FGG axis provides a potential strategy for NSCLC targeted therapy.

## Introduction

Lung cancer is one of the most frequently occurring cancer types and the leading cause of cancer-related death worldwide [1, 2]. According to their clinicopathologic characteristics, lung cancers are broadly classified into two types: small cell lung cancer (SCLC) and non-small cell lung cancer (NSCLC). NSCLC occupies around 85% of total lung cancer cases [3]. Clinical data establish an inverse association between the NSCLC patients overall survival and cancer expression of intercellular adhesion molecule-1 (ICAM-1) [4], a transmembrane glycoprotein belonging to the immunoglobulin (Ig) superfamily of adhesion molecules. NSCLC patients with high neoplastic ICAM-1 expression and serum soluble ICAM-1 levels show more aggressive lung cancer progression [4–6], suggesting a potential role of ICAM-1 in promoting NSCLC.

ICAM-1 (CD54) is a type I transmembrane protein with five extracellular Ig-like domains, which can interact with multiple extracellular and cell surface receptor ligands and plays pleiotropic roles in modulating cell-cell or cell-matrix adhesion and signal transduction under both homeostatic and pathological conditions [7, 8]. ICAM-1 is best known for its role in regulating leukocyte homing from circulation to sites of inflammation via binding to leukocyte integrin αLβ2 (CD11a/CD18, LFA-1) and αMβ2 (CD11b/CD18, Mac-1) through its Ig domain 1 and 3, respectively [8, 9]. In cancer, ICAM-1 has been shown to play a role in recruiting integrin β2-expressing immune cells from bloodstream to ICAM-1–expressing tumor, thus initiating cancer-related immune responses throughout oncogenesis [7, 10, 11]. ICAM-1–β2 interaction also allows tumor cells to adhere to leukocytes, which facilitates the subsequent tumor metastatic process via promoting tumor cell extravasation from vascular endothelium and triggering intracellular signaling to support tumor cell proliferation and migration [12–14]. ICAM-1 can also interact with MUC1, a transmembrane mucin mainly expressed by epithelial malignant cells [15]. It is reported that ICAM-1–MUC1 interaction reprograms cytokine and protease secretion in tumor milieu and induces ICAM-1 up-regulation in malignant cells, thus promoting tumor cell migration and metastasis [16–18]. To date, most of the studies on the role of ICAM-1 in cancer focus on cancer-related immune responses and metastasis, little is known about its intrinsic roles on malignant cell growth.

Besides β2 integrins and MUC1, the plasma protein fibrinogen (FG) is another ligand for ICAM-1. FG is composed of three pairs of nonidentical polypeptide chains (α, β and γ chains) and the γ chain (FGG) binds to ICAM-1 Ig domain 1 [19]. It is reported that the binding of FG to ICAM-1 induces intracellular signalings that regulate physiological cellular functions including cell mitogenesis [20], airway epithelial cell mucin production [21] and cardiomyocyte contractility [22]. Recent studies have revealed the roles of FG and ICAM-1 in orchestrating the immune-cancer cycle, including regulating immune cell effector functions during inflammation [23], as well as manipulating cancer cell metastasis and tumor angiogenesis [24]. FG promotes myelomonocytic cell adhesion to and migration across ICAM-1‒expressing endothelial monolayer to initiate the earliest events of immune responses [23]. In addition, it is reported that plasma FG enhances gallbladder cancer cell metastasis and extravasation through inducing ICAM-1 expression, which is accompanied by intensive pro-angiogenic macrophages recruitment, further facilitating angiogenesis in the tumor microenvironment [24], indicating that FG plays a role in potentiating tumor metastasis and angiogenesis by inducing ICAM-1 expression. Moreover, individual FG α chain (FGA) can interact with integrin α5, which restrains NSCLC cell proliferation, migration and invasion but promotes its epithelial-mesenchymal transition through suppressing AKT-mTOR signaling pathway [25]. However, administration of individual FGA or intact FG to NSCLC cells results in contrasting cell proliferation phenotypes, of which FGA induces cell apoptosis in human lung adenocarcinoma cells but intact FG increases cell proliferation and migration, illustrating an opposite or competitive role of individual FG chains in cancer cell growth. Indeed, FGG is reported to promote tumor cell migration and invasion in hepatocellular carcinoma cells [26], as well as to maintain tumor cell proliferation and disease progression in prostate cancer [27], emphasizing diverse and complex functions of FG components in the tumor microenvironment. Beyond that, the role of neoplastic ICAM-1–FG interaction in regulating malignant tumor cell growth is still largely unknown.

Here, we demonstrated the vital role of neoplastic ICAM-1 in protecting NSCLC cell from apoptosis through the ligation of FGG. Loss of ICAM-1–FGG interaction resulted in caspase-9/3–dependent apoptosis by initiating signaling cascade including the decreased phosphorylation of Akt/ERK1/2 and activation of JNK and p38 MAPK pathways. Antibody specifically blocking ICAM-1‒FGG interaction significantly suppressed NSCLC tumor growth in a mouse xenograft model. Overall, our findings reveal a role of ICAM-1–FGG axis and its underlying mechanism in supporting NSCLC cell survival. Interfering ICAM-1–FGG interaction could be a new strategy for the targeted therapy of NSCLC.

## Results

### Tumor ICAM-1 expression positively associates with poor prognosis in NSCLC

**Fig. 1:**
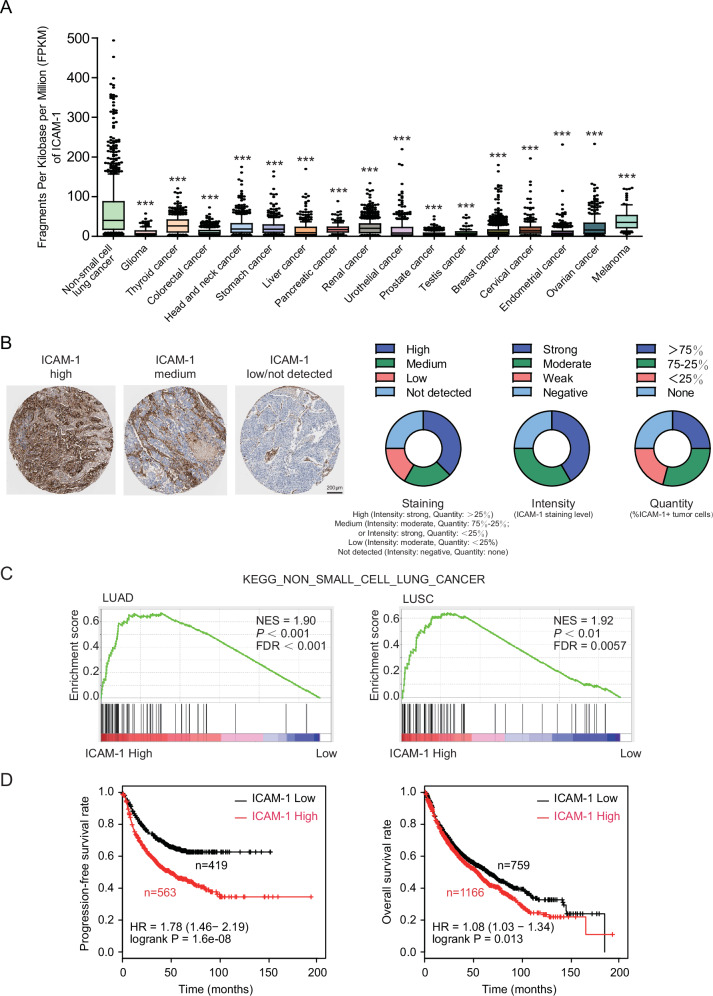
ICAM-1 associates with poor prognosis in human non-small cell lung cancer. **A** Box-and-whiskers plot of ICAM-1 expression profile across different tumor types in TCGA database (https://portal.gdc.cancer.gov/). 10–90 percentile; dots, outliers; midline, median; boundaries, quartiles. One-way ANOVA with Dunnett’s multiple comparisons test. **B** ICAM-1 staining patterns in NSCLC specimens from the Human Protein Atlas (HPA) (http://www.proteinatlas.org/). Left panel, representative images of ICAM-1 high-, medium- and low/not detected-expression NSCLC specimens stained with HPA004877 (anti–ICAM-1) mAb. Scale bar, 200 µm. Right panel, staining, intensity, and quantity plots from the HPA. The annotations *Intensity* and *Quantity* for IHC staining are used to define the ICAM-1 expression level, of which *Intensity* represents the level of antibody staining and *Quantity* represents the fraction of positively stained tumor cells. **C** GSEA analysis for gene expression profiles of NSCLC patients with high or low ICAM-1 expression in the TCGA lung cancer datasets. NES, normalized enrichment score; FDR, false discovery rate. **D** NSCLC patients with high ICAM-1 expression present a worse progression-free survival (*P* < 0.001) and overall survival (*P* < 0.05) compared with patients with low ICAM-1 expression (https://kmplot.com/analysis/). ***P < 0.001.

We first compared ICAM-1 expression level in 17 different cancer types by analyzing the normalized mRNA expression data from The Cancer Genome Atlas (TCGA) database. The results showed that ICAM-1 was significantly highly expressed in NSCLC among a variety of malignancies (Fig. 1A). In addition, lung cancer tissue microarray data from Human Protein Atlas (HPA) database [28] showed consistent results. Among 24 tumor samples from 13 NSCLC cancer patients, the majority of tumors showed medium (staining intensity: moderate, quantity: 75% -25% ; or staining intensity: strong, quantity: <25%) to high (staining intensity: strong, quantity: >25%) immunohistochemistry staining for ICAM-1 (Fig. 1B). Gene set enrichment analysis (GSEA) of the RNA-seq data of 1016 NSCLC patient samples (519 lung adenocarcinoma, LUAD and 497 lung squamous cell carcinoma, LUSC) from TCGA database indicated that tumor tissue ICAM-1 expression level was positively correlated with pathways regulating NSCLC progression (Fig. 1C). We further analyzed ICAM-1 expression in different cell types within the NSCLC tumor microenvironment using a publicly available core NSCLC atlas compiling single-cell RNA sequencing (scRNA-seq) data of 360,038 cells including malignant, epithelial, immune and stromal/endothelial components from 8 studies comprising 159 samples [29] to further explore neoplastic ICAM-1’s weight in NSCLC. Of note, *ICAM1* was dominantly enriched among LUAD and LUSC tumor cells in addition to several alveolar cells, endothelial cells and some macrophages (Supplementary Fig. S1), suggesting the leading functions of tumorous ICAM-1 in regulating NSCLC progression. Moreover, Kaplan-Meier analysis revealed significant association of high ICAM-1 expression with poor progression-free survival (FP) and overall survival (OS) of NSCLC patient cohorts in databases including the Gene Expression Omnibus (GEO), European Genome-phenome Archive (EGA) and TCGA (Fig. 1D) [30]. These results suggest an adverse role of ICAM-1 in NSCLC.

### Loss of ICAM-1 in NSCLC cells triggers cell apoptosis

**Fig. 2:**
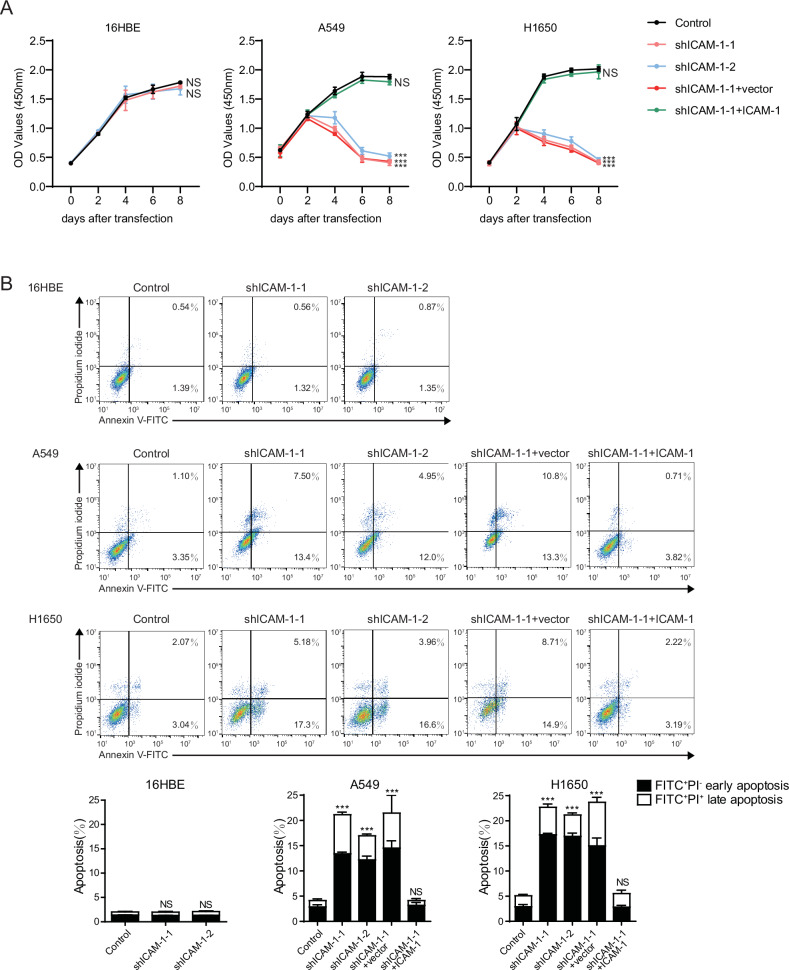
Knockdown of ICAM-1 significantly induces NSCLC cell apoptosis. **A** The viabilities of 16HBE, A549 and H1650 cells with knockdown or re-expression of ICAM-1 were detected by the CCK-8 assay. Data are represented as mean ± SD, n = 3. Two-way ANOVA. **B** Upper panel, flow cytometric analyses of apoptotic cells with knockdown and re-expression of ICAM-1. A representative result of three independent experiments is shown. Living cells tested negative for both Annexin V-FITC and PI. Populations testing FITC^+^/PI^−^ were classified as early-stage apoptotic cells, and double-positive cells were classified as late-stage apoptotic cells. Lower panel, bar graphs quantifying the percentage of early-stage apoptotic and late-stage apoptotic cells. Data are represented as mean ± SD, n = 3. One-way ANOVA with Dunnett’s multiple comparisons test. All data are from three independent experiments. ***P < 0.001; NS, *P* > 0.05.

To investigate the intrinsic roles of ICAM-1 in regulating NSCLC progression, we chose two NSCLC cell lines A549 and NCI-H1650, which showed significant higher levels of ICAM-1 expression than human bronchial epithelial cell line 16HBE (Supplementary Fig. S2), and knocked down ICAM-1 expression with short hairpin RNA (shRNA) in these cells (Supplementary Fig. S3). Notably, knockdown of ICAM-1 in both A549 and H1650 cells led to dramatic viability (CCK-8 OD) reduction (Fig. 2A), which was largely ameliorated by re-expression of ICAM-1. Annexin V and propidium iodide (PI) double staining showed that ICAM-1 knockdown in A549 and H1650 cells induced significant cell apoptosis (Fig. 2B). Both Annexin V-FITC^+^PI^-^ early apoptosis and Annexin V-FITC^+^PI^+^ late apoptosis occurred after ICAM-1 silencing. Re-expression of ICAM-1 in ICAM-1–knockdown A549 and H1650 cells fully prevented cell apoptosis (Fig. 2B). By contrast, ICAM-1 knockdown did not affect 16HBE cell viability and apoptosis (Fig. 2A, B). Thus, ICAM-1 has an essential role in supporting NSCLC cell survival but is not required for normal bronchial epithelial cell growth. Moreover, direct overexpression (O/E) of ICAM-1 in A549 and H1650 cells showed no effects on cell apoptosis (Supplementary Fig. S4).

### ICAM-1 supports NSCLC cell survival via interacting with cancer cell-derived fibrinogen γ chain

**Fig. 3:**
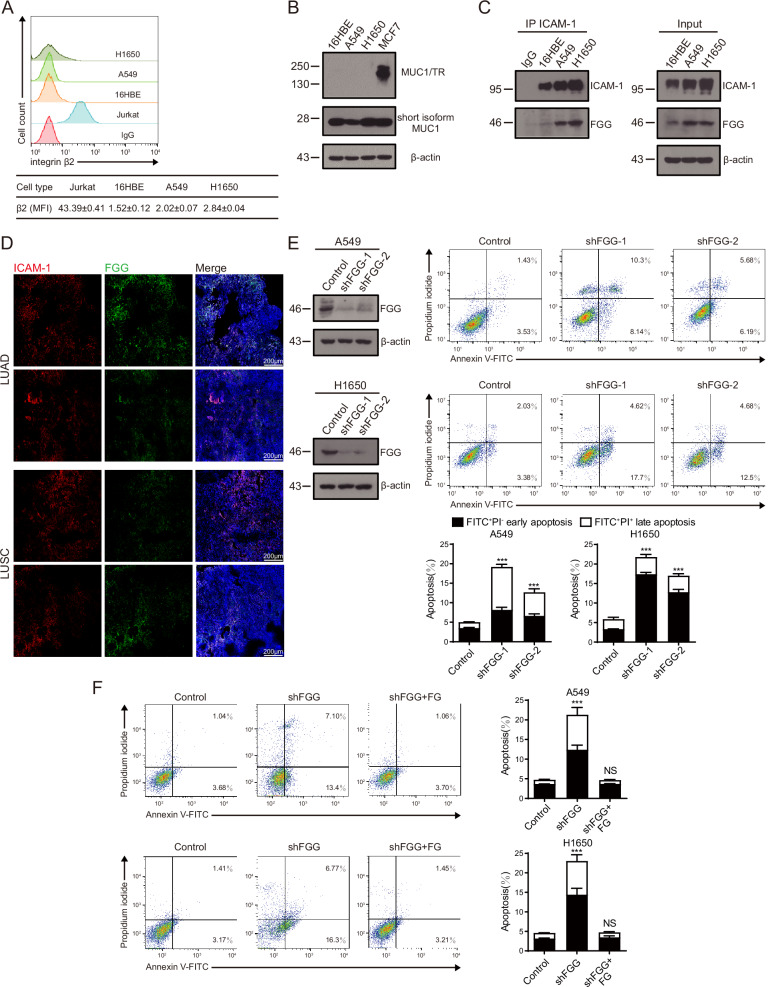
Expression of ICAM-1 ligands in NSCLC cells. **A** Flow cytometry analysis of integrin β2 expression on 16HBE, A549 and H1650 cells. Jurkat cells were used as a positive control. The numbers in the table show the specific mean fluorescence intensities of TS1/18 (anti–β2) mAb. **B** Expression levels of ICAM-1–binding TR domain of MUC1 were determined by immunoblotting in 16HBE, A549 and H1650 cells. MCF7 cells were shown as a positive control. **C** Co-immunoprecipitation of FGG with ICAM-1 in 16HBE, A549 and H1650 cells. **D** Representative images of NSCLC tumors (patient no. 423, 406, LUAD; patient no. 416, 290, LUSC) stained with anti–ICAM-1 and anti-FGG antibodies. Nuclei were counterstained with DAPI. Scale bar, 200 µm. **E** Left panel, expression levels of FGG were determined by immunoblotting in A549 and H1650 cells with knockdown of FGG. Right panel, flow cytometric analyses of apoptotic cells with knockdown of FGG. **F** Flow cytometric apoptosis analyses of FG-treated FGG-knockdown NSCLC cells. A representative result of three independent experiments is shown. Bar graphs quantifying the percentage of early-stage apoptotic and late-stage apoptotic cells. Data are represented as mean ± SD, n = 3. One-way ANOVA with Dunnett’s multiple comparisons test. All data are from three independent experiments. ***P < 0.001; NS, *P* > 0.05.

To explore the mechanism underlying ICAM-1 deficiency-induced NSCLC cell apoptosis, we examined the expression of ICAM-1 ligands, including integrin β2, MUC1 and FGG, in A549 and H1650 cells. FACS analysis showed that NSCLC cells did not express integrin β2 compared with Jurkat cells as a positive control (Fig. 3A). In contrast to MUC1-expressing MCF7 control cells, A549 and H1650 cells showed no expression of ICAM-1–binding MUC1 TR domain [17] (Fig. 3B). Only MUC1 short isoform was detected, which does not bind to ICAM-1. Notably, we found a strong association between ICAM-1 and FGG in A549 and H1650 cells (Fig. 3C). Beyond that, confocal microscopy with double-staining of NSCLC LUAD and LUSC specimens demonstrated obvious expression and colocalization of ICAM-1 and FGG on malignant cells (Fig. 3D). Similar to ICAM-1, the increased FGG expression was also associated with worse long-term progression-free and overall survival outcomes in NSCLC patients according to Kaplan-Meier estimate (Supplementary Fig. S5A). Next, we analyzed the TCGA database to determine whether the expression level of ICAM-1 was correlated with FGG [31]. We found that FGG was positively correlated with ICAM-1 expression in NSCLC data sets (Supplementary Fig. S6A). Knockdown of FGG resulted in A549 and H1650 cell apoptosis similar to that induced by ICAM-1 knockdown (Fig. 3E). In addition, administration of FG protein to FGG-knockdown NSCLC cells prominently prevented cell apoptosis (Fig. 3F), indicating that intact FG exhibits anti-apoptosis effects to NSCLC cells. These results encourage us to hypothesize that ICAM-1–FGG interaction may play a vital role in supporting NSCLC cell survival.

**Fig. 4:**
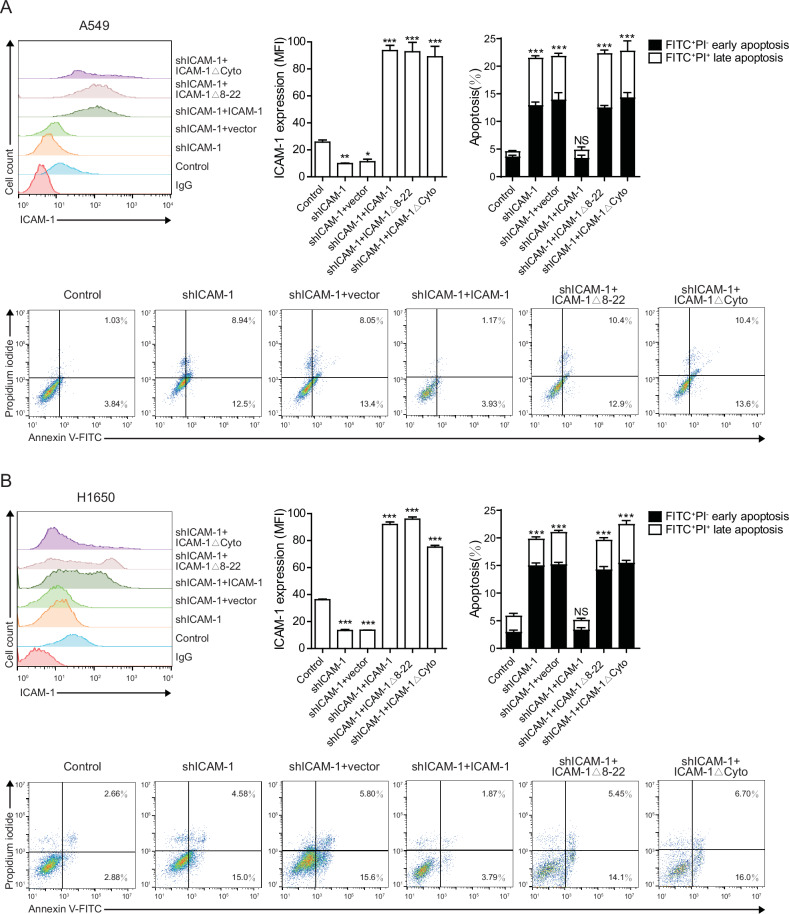
ICAM-1 interacting with FGG suppresses NSCLC cell apoptosis. A549 and H1650 cells with ICAM-1 knockdown and re-expression of WT ICAM-1, ICAM-1 mutant (ICAM-1Δ8-22) or ICAM-1 cytoplasmic domain truncation mutant (ICAM-1ΔCyto) were analyzed in (**A**) and (**B**), respectively. Left panel, flow cytometry analyses of ICAM-1 surface expression on the indicated cells. Middle panel, quantification of ICAM-1 expression. Right panel, cell apoptosis was examined by Annexin V-FITC/PI staining assay. Dot plots for flow cytometric analyses of apoptotic cells are shown in the lower panel. A representative result of three independent experiments is shown. Data are represented as mean ± SD, n = 3. One-way ANOVA with Dunnett’s multiple comparisons test. All data are from three independent experiments. *P < 0.05; **P < 0.01; ***P < 0.001; NS, *P* > 0.05.

To verify our hypothesis, we established an ICAM-1 mutation (ICAM-1Δ8-22), which lacks the FGG-binding motif Lys8-Ser22 located in the Ig domain 1, to abolish ICAM-1–FGG interaction [20, 32]. Re-expression of WT ICAM-1 but not ICAM-1Δ8-22 mutant in ICAM-1–knockdown A549 and H1650 cells ameliorated the ICAM-1 knockdown-induced cell apoptosis (Fig. 4A, B), indicating the ICAM-1–FGG interaction is essential to the survival of NSCLC cells. Similarly, re-expression of ICAM-1 cytoplasmic domain deletion mutant (ICAM-1ΔCyto) in ICAM-1–knockdown A549 and H1650 cells did not rescue the cell apoptotic phenotype (Fig. 4A, B), suggesting that the binding of FGG to ICAM-1 may activate an anti-apoptosis signaling in NSCLC cell through ICAM-1 intracellular signaling.

### Disruption of ICAM-1–FGG axis activates apoptotic pathways in NSCLC cells

**Fig. 5:**
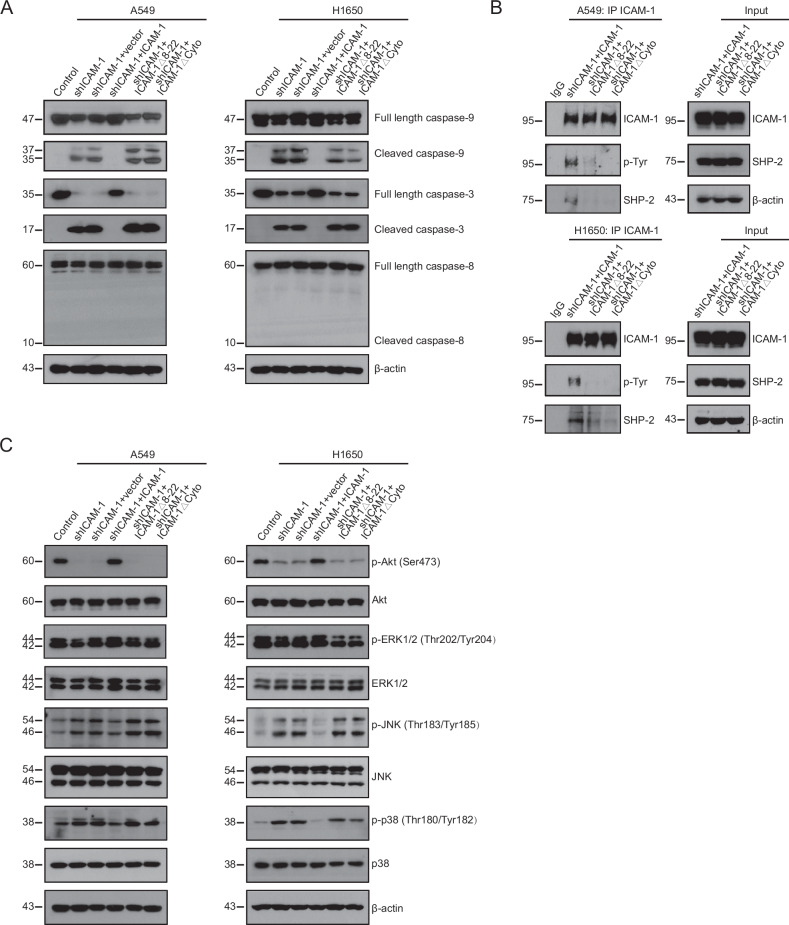
ICAM-1 knockdown activates apoptotic pathways in NSCLC cells. **A** Immunoblotting of caspases in A549 and H1650 with ICAM-1 knockdown and re-expression of WT ICAM-1, ICAM-1Δ8-22 or ICAM-1ΔCyto. **B** Tyrosine phosphorylation of ICAM-1 and co-immunoprecipitation of SHP-2 with ICAM-1 in the indicated cells. **C** Phosphorylation of Akt (Ser473), ERK1/2 (Thr202/Tyr204), JNK (Thr183/Tyr185) and p38 (Thr180/Tyr182) in A549 and H1650 cells in the indicated cells. Total Akt, ERK1/2, JNK, p38 and β-actin are shown in the lower rows. A representative result of three independent experiments is shown.

The caspase family of proteases plays crucial roles in the initiation, regimen and amplification of intracellular apoptotic signals [33]. Initiator caspases such as caspase-8 and -9 activate executioner caspases including caspase-3, -6 and -7 that subsequently orchestrate their activities to execute cell death [34]. We next investigated whether loss of ICAM-1–FGG ligation modulated the activation of caspases. Knockdown of ICAM-1 in A549 and H1650 cells resulted in the cleavage of caspase-9 but not caspase-8, and the consequent activation of caspase-3 (Fig. 5A). Re-expressing WT ICAM-1 fully suppressed the ICAM-1 knockdown-induced cleavage of caspase-9/3 (Fig. 5A). However, overexpression of ICAM-1Δ8-22 or ICAM-1ΔCyto mutant was unable to inhibit the activated caspase-9/3 signaling (Fig. 5A), indicating ICAM-1–FGG ligation and ICAM-1 intracellular signaling are crucial for the shut-down of caspase-9/3 signaling in NSCLC cells. Thus, ICAM-1–FGG interaction provides an anti-apoptosis signal for NSCLC cells via ICAM-1 intracellular pathways.

Src homology domain 2 (SH2)-containing phosphatase-2 (SHP-2) can associate with the phosphorylated cytoplasmic domain of ICAM-1 [35, 36] and restrict cell apoptosis via inhibition of caspase-9 and -3 cleavage [37]. A strong association between SHP-2 and tyrosine-phosphorylated WT ICAM-1 was detected in A549 and H1650 cells, whereas FGG-binding deficient ICAM-1Δ8-22 mutant showed nearly no tyrosine phosphorylation and undetectable binding of SHP-2 (Fig. 5B). As expected, ICAM-1 lacking cytoplasmic domain (ICAM-1ΔCyto) showed no tyrosine phosphorylation and SHP-2 binding (Fig. 5B). These data suggest FGG–ICAM-1 ligation induces ICAM-1 phosphorylation and promotes its association with SHP-2.

We next examined the activation of c-Jun N-terminal kinase (JNK) and p38 MAPK that is associated with cell apoptosis [38–40] and the phosphorylation of extracellular signal-regulated kinase (ERK) 1/2 and Akt, which are reported to be activated by SHP-2 and facilitate cell survival and proliferation [41, 42]. Knockdown of ICAM-1 in A549 and H1650 cells promoted the phosphorylation of JNK and p38 but suppressed phosphorylation of ERK1/2 and Akt (Fig. 5C). These changes were restored by re-expressing WT ICAM-1 in the cells, but not by re-expression of ICAM-1Δ8-22 or ICAM-1ΔCyto (Fig. 5C). These data indicate that ICAM-1–FGG axis sustains cell survival-related ERK and Akt signaling but restrains apoptosis-related JNK and p38 pathways, thus protects NSCLC cells from apoptosis.

### Disrupting ICAM-1–FGG interaction suppresses tumor growth in vivo

**Fig. 6:**
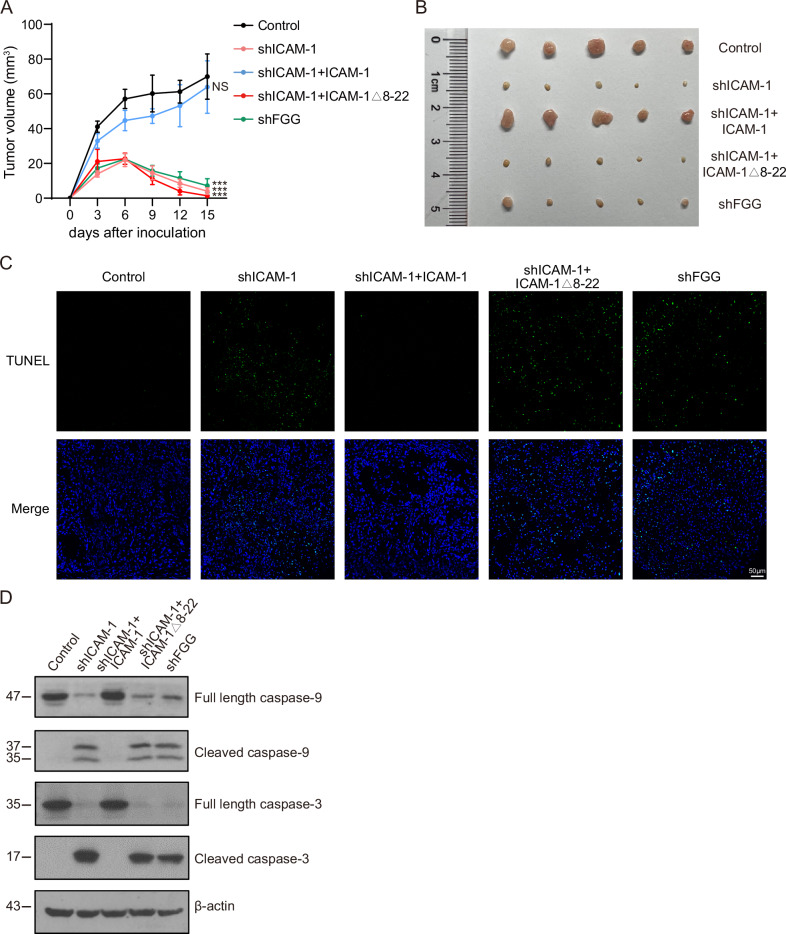
Inhibition of ICAM-1–FGG interaction suppresses NSCLC tumor growth in mice. Female BALB/c nude mice were subcutaneously inoculated with 2×10^6^ WT and modified A549 cells. **A** Tumor volumes were measured and calculated every 3 days. Data are represented as mean ± SEM, n = 6 mice (Control, shICAM-1, shICAM-1 + ICAM-1), n = 5 mice (shICAM-1 + ICAM-1Δ8–22), n = 7 mice (shFGG). Two-way ANOVA. All data are from three independent experiments. ***P < 0.001; NS, *P* > 0.05. **B** Tumors dissected on day 15 and imaged as shown. **C** TUNEL staining of tumor tissues from the indicated groups. Scale bar, 50 µm. **D** Immunoblotting of caspase-9/3 in tumor tissues from the indicated groups. A representative result of three independent experiments is shown.

To further validate the role of ICAM-1–FGG axis in regulating NSCLC cell survival in vivo, A549 xenograft model was established by injecting A549 cells subcutaneously into nude mice (Fig. 6A). Animals were monitored for tumor progression every 3 days and euthanized on day 15 after tumor cell inoculation to collect tumor specimens. Compared with A549 cells transfected with control scrambled shRNA (control), tumors from mice receiving ICAM-1 shRNA (shICAM-1)- or FGG shRNA (shFGG)-treated A549 cells showed significantly decreased ICAM-1 or FGG expression (Supplementary Fig. S7). A remarkable slowdown in tumor growth was observed in shICAM-1 and shFGG groups compared with control group (Fig. 6A). Notably, tumors of shICAM-1 and shFGG groups grew slower and displayed spontaneous regression on day 6, followed by a quick and significant shrink in tumor volume in next days (Fig. 6A, B), indicating the death of tumor cells when ICAM-1 or FGG was absent. On day 15, the tumor volumes of shICAM-1 and shFGG groups were significantly smaller than those of control mice (Fig. 6B). Notably, disrupting the ICAM-1–FGG interaction using ICAM-1Δ8-22 mutation showed similar tumor inhibition as knockdown of ICAM-1 or FGG (Fig. 6A, B). In line with these results, tumors of shICAM-1, shFGG and shICAM-1 + ICAM-1Δ8-22 groups showed strong cell apoptosis signals in TUNEL staining (Fig. 6C) and activation of caspase-9/3 apoptotic signaling (Fig. 6D). Re-expression of WT ICAM-1 in ICAM-1 knockdown cells efficiently suppressed cell apoptosis and rescued tumor growth in mice (Fig. 6A–D). These in vivo data reinforce the mechanism that ICAM-1 supports NSCLC cell survival via interacting with cancer cell-derived FGG.

### ICAM-1 blocking antibody inhibits NSCLC tumor growth

**Fig. 7:**
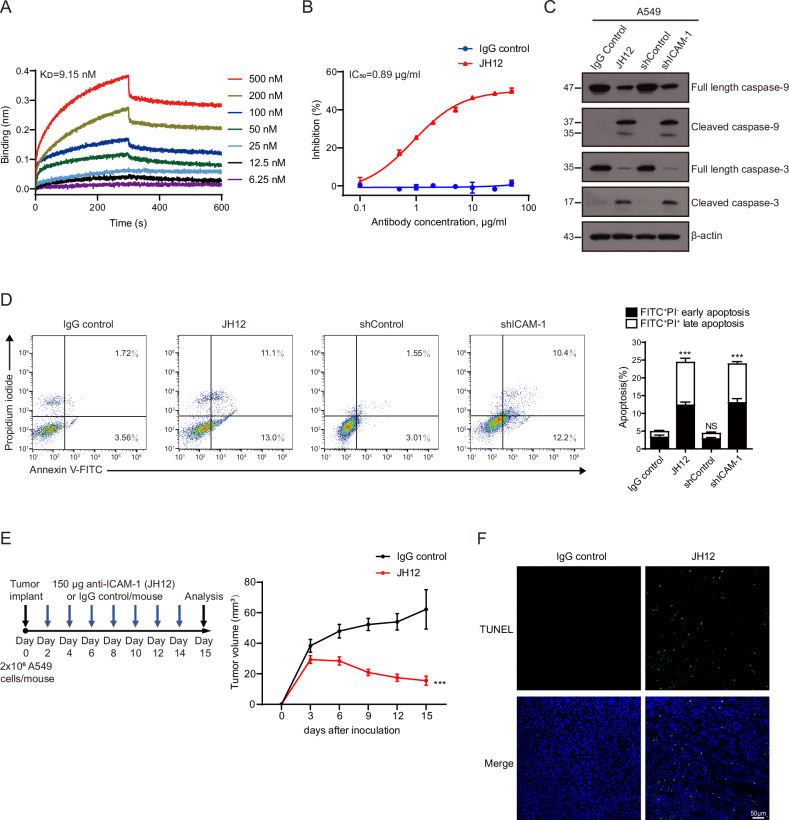
ICAM-1 blocking antibody JH12 effectively suppresses tumor growth in A549 xenograft model. **A** Binding of JH12 antibody to ICAM-1 D1D2 was measured by Bio-Layer Interferometry assay. **B** Flow cytometry analysis of the binding of FG protein to A549 cells in the presence of antibody JH12 or human IgG control. Data are represented as mean ± SD, n = 3. **C** Immunoblotting of caspase-9/3 in A549 cells treated with JH12, human IgG control or ICAM-1 shRNA. A representative result of three independent experiments is shown. **D** Apoptosis of the indicated A549 cells was examined by Annexin V-FITC/PI staining assay. Dot plots for flow cytometric analyses of apoptotic cells are shown in the left panel. A representative result of three independent experiments is shown. Data are represented as mean ± SD, n = 3. One-way ANOVA with Dunnett’s multiple comparisons test. **E** Left panel, therapy regimen. Right panel, tumor volumes in IgG control and JH12-treated mice. Data are represented as mean ± SEM, n = 10 mice (IgG control), n = 20 mice (JH12). Two-way ANOVA. All data are from three independent experiments. **F** TUNEL staining of A549 xenografts from IgG control and JH12-treated mice. Scale bar, 50 µm. A representative result of three independent experiments is shown. ***P < 0.001; NS, *P* > 0.05.

To pursue a strategy of inducing NSCLC cell apoptosis by blocking ICAM-1‒FGG interaction, we screened monoclonal antibodies (mAbs) against FGG-binding motif in ICAM-1 using single-chain fragment variable (scFv) phage display library. Among the identified mAbs that recognize ICAM-1 Lys8-Ser22 motif, clone JH12 bound to ICAM-1 at nanomolar affinity with an equilibrium dissociation constant of 9.15 nM (Fig. 7A). JH12 mAb treatment efficiently inhibited FG binding to A549 cells with a half maximal inhibitory concentration (IC_50_) of 0.89 μg/ml (Fig. 7B) and significantly induced caspase-9/3 cleavage and A549 cell apoptosis (Fig. 7C, D). Moreover, JH12 mAb did not affect soluble ICAM-1 binding to integrin β2-expressing NK-92 cells (Supplementary Fig. S8), indicating JH12 mAb does not affect ICAM-1‒integrin β2 interaction.

We next assessed the therapeutic effect of JH12 mAb on NSCLC tumor in vivo. Mice were intraperitoneally injected with JH12 mAb or isotype-matched IgG control every two days until the end of the experiment (Fig. 7E). Administration of JH12 mAb significantly restrained tumor growth (Fig. 7E) and induced cancer cell apoptosis (Fig. 7F) compared with IgG control group. These results demonstrate that blocking ICAM-1‒FGG interaction by JH12 mAb effectively suppresses NSCLC tumor growth in vivo by inducing tumor cell apoptosis.

The RAS-RAF-MAPK ERK kinase (MEK)-ERK pathway is a major determinant in the control of diverse cellular processes such as proliferation, survival, differentiation and motility, which controls cell survival and proliferation in NSCLC [43]. Ectopic ERK1/2 activation contributes to NSCLC, and as such represents an attractive target for the development of anticancer drugs [44]. Considering interference of ICAM-1‒FGG axis resulted in the suppressed ERK1/2 phosphorylation (Fig. 5C) and subsequent NSCLC cell apoptosis (Fig. 7C, D), we wondered whether blocking ICAM-1‒FGG interaction using JH12 mAb has synergistic effects in combination with recently developed potent ERK pathway small-molecule inhibitors. We examined A549 cell apoptosis treated with JH12 mAb in the presence or absence of SCH772984 [45], a novel specific ERK inhibitor which can potently inhibit ERK1 and ERK2 activity. JH12 plus SCH772984 treatment induced more caspase-9/3 cleavage and A549 cell apoptosis compared with JH12 mAb or SCH772984 administrated alone (Supplementary Fig. S9), suggesting ICAM-1 blocking antibody exhibits a synergistic effect with ERK inhibitor. Thus, ICAM-1‒FGG inhibitor may function as a promising therapeutic co-target in combination with other targeted drugs in cancer therapy.

In summary, our study demonstrates that ICAM-1–FGG ligation provides an anti-apoptosis signaling for NSCLC cells. Engagement of FGG with ICAM-1 on NSCLC cells promotes ICAM-1 phosphorylation and its association with SHP-2 and induces the activation of anti-apoptotic signaling including Akt and ERK1/2. Disruption of ICAM-1–FGG interaction suppresses anti-apoptotic signaling and consequently activates caspase-9/3 pathway, leading to cancer cell death. Thus, ICAM-1–FGG axis may be a promising target for NSCLC targeted therapy.

## Discussion

It is well-known that ICAM-1 plays a crucial role in mediating cancer cell migration and spreading [7, 46, 47], however little is known about tumorous ICAM-1 intrinsic effects with regards to cancer cell survival and tumor progression. In this study, we demonstrate that cell surface-expressing ICAM-1 provides anti-apoptotic signal to NSCLC cells through interacting with cancer cell-derived FGG. Disrupting ICAM-1‒FGG interaction prominently induces NSCLC cell apoptosis thus restraining tumor progression.

During tumor progression, leaky vessels are often found in bands of stroma interposed between individual tumor nodules and are present in highest concentration at the tumor-host interface at the edge of the tumor [48]. This leaky nature of tumor blood vessels leads to the deposition of vascular proteins in the tumor matrix. Among them, FG, a soluble vascular protein, is deposited in the tumor matrix [49]. Then extravasated soluble FG rapidly forms an insoluble clot or gel on conversion to fibrin by the action of the serine protease thrombin, which is activated by a cascade of enzymatic reactions triggered by vessel wall injury, activated blood cells, or the prothrombogenic activity of tumor cells [48–50]. This crosslinked fibrin gel, constituting the principal ingredient of blood clot, is difficult to interact with neoplastic ICAM-1 due to its insoluble properties and resulting steric hindrance, suggesting that NSCLC cell-derived FGG may be sufficient to execute its anti-apoptosis functions when associates with ICAM-1.

In addition to tumor cell producing or leaky blood vessel deposited FGG, other non-tumor cell expressing FGG may also participate in ICAM-1‒FGG mediated NSCLC cell survival. To investigate the predominant FGG-expressing cell type during NSCLC progression, we analyzed lung cancer tissue microarray data from HPA database. Results showed that among 6 FGG staining positive NSCLC tumor specimens, the majority of samples (83.3%) exhibited tumor cell specific expression of FGG. Only 1 specimen was observed FGG staining not only on tumor cells but also on stroma cells (Supplementary Fig. S6B), indicating that FGG is predominantly produced by NSCLC tumor cells and is sufficient to maintain malignant cell survival during NSCLC progression.

FG, its components, and their derivatives appear to play different roles in the tumor microenvironment [25, 51]. Although a previous study has revealed that FGA suppresses tumor growth and metastasis through association with integrin α5, the intact FG protein or FGG unlikely has similar effects on LUAD survival [25], which is consistent with our findings that tumor cell derived-FGG executes its anti-apoptosis functions when ligation to NSCLC cell surface-expressing ICAM-1. Gene knockout (KO) for different chains of the FG molecule is now warranted to elucidate their specific roles in pathology of various organs. However, mutations within the FG structural genes (primarily *FGA*) that eliminate any part of the coding sequence, alter transcription, mRNA splicing or stability, and protein synthesis, assembly, or secretion directly associate with FG disorders [52]. *FGA* or *FGG* KO in mice leads to precluded assembly of functional circulating FG protein, resulting in congenital hypofibrinogenemia and afibrinogenemia as well as related atherosclerosis, colitis, multiple sclerosis and pregnancy failure [53, 54]. And mice carrying the *FGG*^*390-396A*^ mutation, of which FG has been mutated to lack the integrin αMβ2 binding motif but retained normal clotting function, exhibit affected blood-induced myeloid cell functions resembling FG-deficient phenotypes in *FGA*^-/-^ mice [55–57], suggesting genetically engineering of individual FG chains contributes to FG-mediated cell function loss. Moreover, currently in tumor cells, the expression and regulation of FG and its polypeptide chains are not sufficiently understood. It has been reported that the dysregulation of FGG but not other chains of FG frequently occurs in many malignant cancer types [26, 27], emphasizing that FGG may function beyond the structural domain of FG in the tumor environment.

Both ICAM-1 and FGG expression were negatively correlated with OS rate not only in NSCLC patients (Fig. 1D and Supplementary Fig. S5A) but also in a group of patients across several cancer types including colorectal, pancreatic and renal cancers (Supplementary Fig. S5B) [58], indicating that a subset of human malignancies may experience the heightened ICAM-1–FGG signaling. It’s prospective to attenuate ICAM-1–expressing lung cancer progression as well as other ICAM-1‒expressing solid tumor growth by blocking ICAM-1–FGG ligation. According to our preliminary data, several cancer cell lines derived from diverse tumor origins including colorectal adenocarcinoma, pancreatic ductal adenocarcinoma and renal cell carcinoma showed cell apoptosis when ICAM-1 expression was abrogated (Supplementary Fig. S10), which emphasizes ICAM-1–FGG signaling as a general target for treatment of multiple cancer types. It’s noteworthy that ICAM-1 and FGG expression is correlated with favorable prognostic in particular malignant tumors such as breast and liver cancer, suggesting diverse roles of ICAM-1–FGG signaling in different malignant cell development.

FGG and integrin β2 bind to distinct binding sites in ICAM-1 [9, 59], shedding light on the possibility of exploiting neutralizing antibodies or compounds to specifically obstruct ICAM-1‒FGG interaction without affecting integrin β2‒ICAM-1 binding involved in anti-tumor immune responses. In this study, we successfully developed JH12 mAb that specifically recognizes ICAM-1 FGG-binding motif and strongly blocked ICAM-1 binding to FGG without affecting integrin β2 interaction (Supplementary Fig. S8B). Synthetic peptide with an amino acid sequence matching FGG-binding motif in ICAM-1 (ICAM-1 Lys8-Ser22) has been shown to block FGG-induced various biological processes [20, 21]. However, ICAM-1 Lys8-Ser22 peptide failed to induce NSCLC cell apoptosis (data not shown), probably due to excess autocrine FGG secreted by lung cancer cells which cannot been fully neutralized by the peptide.

Previous studies have shown that cell apoptosis is associated with the suppressed ERK activation and the upregulated JNK and p38 signaling [60–63], which is in line with our observations that down-regulation of p-ERK1/2 and up-regulation of p-JNK/p38 MAPK resulted in NSCLC cell apoptosis. Akt is reported to collaborate contradictorily with JNK and p38 MAPK pathways to inhibit cell apoptosis [64, 65]. Moreover, SHP-2 has been demonstrated to activate Akt pathway and thus triggering anti-apoptotic signaling by suppressing caspase-3 mediated apoptosis [66]. In our study, we found that ICAM-1 knockdown regulated the phosphorylation of SHP-2, Akt, JNK and p38 in NSCLC cells (Fig. 5B, C), suggesting a crosstalk between ICAM-1 and intracellular signaling cascades that orchestrates the lung cancer cell survival signals.

**Fig. 8:**
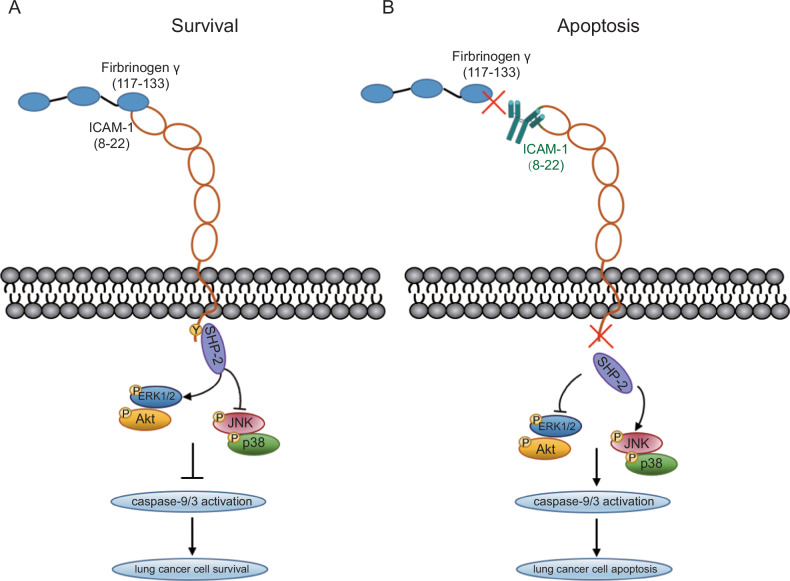
ICAM-1–FGG interaction provides anti-apoptotic signal to NSCLC cells. **A** ICAM-1 is tyrosine phosphorylated upon ligation with FGG through motif Lys8-Ser22, followed by association with SHP-2, activation of ERK1/2 and Akt and restraint of JNK and p38 MAPK signaling, and consequently inhibits caspase-9/3 activation and prevents cell apoptosis. **B** Abolishing ICAM-1–FGG interaction disrupts the anti-apoptotic signaling and induces cancer cell death.

Taken together, our study demonstrates that FGG binds to ICAM-1 on NSCLC cell surface and triggers anti-apoptotic signaling in cancer cells, therefore protects cancer cell from apoptosis (Fig. 8A). Blocking ICAM-1–FGG interaction disrupts the survival signaling and activates caspase-9/3, thus inducing cancer cell apoptosis and prevents lung cancer progression in vivo (Fig. 8B). Targeting ICAM-1–FGG interaction could be a new strategy for the targeted therapy for NSCLC.

## Materials and methods

### Antibodies and reagents

The antibodies used for flow cytometry were anti-human ICAM-1 (555510, BD Biosciences; ab2213, Abcam), Alexa Fluor 647-conjugated goat anti-mouse IgG (A21236, Invitrogen), FITC-conjugated goat anti-mouse IgG (405305, Biolegend), FITC-conjugated anti-human CD18 (302105, Biolegend), APC-conjugated anti-human CD11a (550852, BD Biosciences), APC-conjugated anti-human CD11b (101212, Biolegend) and APC-conjugated anti-DYKDDDDK Tag (637308, Biolegend). Antibodies against human MUC1 (4538, Cell signaling; ab45167, Abcam), ICAM-1 (HPA002126, Sigma-Aldrich), fibrinogen γ chain (AV44300, Sigma-Aldrich), Caspase-3 (9662, Cell signaling), Cleaved Caspase-3 (9661, Cell signaling), Caspase-8 (4790, Cell signaling), Caspase-9 (9502, Cell signaling), SHP-2 (D50F2) (3397, Cell signaling), mouse Anti-Phosphotyrosine (610000, BD Biosciences), Akt (9272, Cell signaling), Phospho-Akt (Ser473) (4060, Cell signaling), p44/42 MAPK (Erk1/2) (9102, Cell signaling), Phospho-p44/42 MAPK (Erk1/2) (Thr202/Tyr204) (9101, Cell signaling), SAPK/JNK (9252, Cell signaling), Phospho-SAPK/JNK (Thr183/Tyr185) (4668, Cell signaling), p38 MAPK (8690, Cell signaling), Phospho-p38 MAPK (Thr180/Tyr182) (4511, Cell signaling), mouse monoclonal anti–β-actin (AC004, ABclonal) were used to detect the target proteins by immunoblotting. The antibodies used for immunofluorescence staining were anti-FGG (abs111040, absin) and Alexa Fluor 647-conjugated goat anti-rabbit IgG (A21245, Invitrogen). DAPI (D1306) was from Invitrogen. Cell Counting Kit-8 (CCK-8) (C0038) and One Step TUNEL Apoptosis Assay Kit (C1086) were from Beyotime. Annexin V-FITC/PI Apoptosis Detection Kit (A211-02) was from Vazyme. Synthetic ICAM-1 Lys8-Ser22 peptide (KVILPRGGSVLVTCS) was from Genscript. Fibrinogen from human plasma (F3879) was from Sigma. Human IgG Isotype Control (31154) was from Invitrogen. SCH772984 (S7101) was from Selleck.

### cDNAs and cell lines

cDNAs of human ICAM-1, ICAM-1Δ8-22 and ICAM-1ΔCyto were constructed in vector pCDH-puro (Invitrogen). Human bronchial epithelial cell line 16HBE, NSCLC cell line A549 and pancreatic adenocarcinoma cell line PANC-1 were cultured in Dulbecco’s modified Eagle’s medium supplemented with 10% fetal bovine serum, 2 mM glutamine, penicillin (100 U/ml) and streptomycin (100 μg/ml). NSCLC cell line NCI-H1650, colon adenocarcinoma cell line SW480 and renal cell carcinoma cell lines 769-P and 786-O were cultured in RPMI-1640 medium supplemented with 10% fetal bovine serum, 2 mM glutamine, penicillin (100 U/ml) and streptomycin (100 μg/ml). Colon adenocarcinoma cell lines HT-29 and HCT 116 were cultured in McCoy’s 5A medium supplemented with 10% fetal bovine serum, 2 mM glutamine, penicillin (100 U/ml) and streptomycin (100 μg/ml). Nature killer cell line NK-92 was cultured in MEMα medium supplemented with 20% fetal bovine serum, 0.2 mM inositol, 0.1 mM β-mercaptoethanol, 0.02 mM folic acid, 100 U/mL human recombinant IL-2, 2 mM glutamine, penicillin (100 U/ml) and streptomycin (100 μg/ml). All cell lines were purchased from ATCC (Manassas, VA) and tested for mycoplasma contamination negative.

### Gene set enrichment analysis (GSEA)

The Cancer Genome Atlas (TCGA) NSCLC datasets containing gene level count data from RNA-seq and clinical data of primary LUAD and LUSC tumor samples were accessed to identify the association of ICAM-1 with NSCLC-related gene signatures using GSEA 4.1.0 software. Bioconductor package using “KEGG_non-small cell lung cancer” gene set in Molecular Signatures Database (MSigDB) collections [67] was measured. The normalized enrichment score (NES), false discovery rate (FDR) and P value were considered key statistics in GSEA. A gene set was considered significantly enriched when P value was less than 0.05 and FDR was less than 0.25.

### Flow cytometry

Flow cytometry was done as described [68]. 16HBE, A549, H1650, Jurkat and NK-92 cells were stained with antibodies against ICAM-1, integrin β2, αL or αM and then measured using FACSCelesta (BD Biosciences). Data were analyzed using FlowJo V10 software.

### Silencing of ICAM-1 and fibrinogen γ chain expression in lung cancer cell lines

Silencing of human ICAM-1 or fibrinogen γ chain expression in lung cancer cell lines was achieved by shRNA. Cells were infected with recombinant lentivirus, which expressed shRNAs targeting ICAM-1 (shICAM-1–1: 5′-GGAGCAAGACTCAAGACATGA-3′; shICAM-1–2: 5′-CCGGTATGAGATTGTCATCAT-3′) or fibrinogen γ chain (shFGG-1: 5′-CCGGTGGTATTCCATGAAGAA-3′; shFGG-2: 5′-CTGCATCTTAGATGAAAGAT-3′). The oligonucleotide targeting sequence for control scrambled shRNA was 5′-CCTAAGGTTAAGTCGCCCTCG-3′. Silencing of ICAM-1 or fibrinogen γ chain was confirmed by immunoblot 48 h after transfection.

### Cell viability detection

Cells (5000 cells per well) were inoculated into the 96-well plate. 20 μl CCK-8 solution was added to each well and incubated for 2 h. The absorbance at 450 nm was determined by a microplate reader (Multiskan MK3, Thermo Scientific). The experiment was set up in triplicate for statistical significance.

### Cell apoptosis detection

Cell apoptosis was determined by Annexin V-FITC and propidium iodide (PI) double staining using an Annexin V-FITC/PI Apoptosis Detection Kit according to the manufacturer’s instructions. Cells were harvested with EDTA-free trypsin and washed twice with ice-cold phosphate-buffered saline. Then, the cells were resuspended in 100 μl binding buffer plus 5 μl Annexin V-FITC and 5 μl PI staining solution for 10 min at room temperature, followed by flow cytometry analysis.

For fibrinogen treatment, FG protein from human plasma (10 μg/mL) was added into the culture medium of A549 and H1650 FGG-knockdown cells. For JH12 mAb and ERK1/2 inhibitor SCH772984 treatment, anti–ICAM-1 (JH12), human IgG isotype control antibody (20 μg/mL) or DMSO vehicle control, SCH772984 (0.5 μM) were administrated to A549 cells in the serum-free medium for 24 h before flow cytometry analysis.

### Western blot

After washing with ice-cold Tris-buffered saline (TBS) (20 mmol/L Tris-HCl, 150 mmol/L NaCl, pH 7.4), 16HBE, A549, H1650 and MCF7 cells as well as tumor specimens were homogenized and lysed with 100 μl of lysis buffer (TBS containing 1% Triton X-100, 0.05% Tween 20, Complete Protease Inhibitor Cocktail Tablets, and PhosSTOP Phosphatase Inhibitor Cocktail Tablets) for 30 min on ice. Cell lysates were prepared by centrifuging for 15 min at 20,000 × *g*. Supernatants were fractionated by reducing SDS-PAGE.

For co-immunoprecipitation, cells were treated with 5 mM DTBP (Thermo Fisher Scientific) for 30 min, and then lysed with lysis buffer for 30 min on ice. The lysates were then immunoprecipitated with the indicated antibodies. Rabbit IgG was used as a control. β-actin was detected by immune blot as a loading control.

### Immunofluorescence staining

Pathologically confirmed, formalin-fixed, paraffin-embedded (FFPE) human lung adenocarcinoma and lung squamous cell carcinoma biopsy specimens from Fudan University Shanghai Cancer Center, collected between 2007 and 2017, were used for ICAM-1 and FGG staining. All patients gave written informed consent. Sections were cut at a thickness of 5 μm, deparaffinized in xylene and rehydrated through a graded series of ethanol solutions (100%, 95% and 75%). Then, slides were treated by microwave to induce antigen retrieval using citric acid solution for 15 min. The sections were blocked in TBS containing 0.1% Triton X-100, 2% bovine serum albumin (BSA) and 5% normal goat serum for 1 h at room temperature. Then sections were incubated with primary antibody overnight at 4 °C (ICAM-1, 1:200, ab2213, Abcam; FGG, 1:100, abs111040, absin), followed by incubation with corresponding secondary antibodies for 1 h at room temperature (FITC-conjugated goat anti-mouse IgG, 405305, Biolegend; Alexa Fluor 647-conjugated goat anti-rabbit IgG, A21245, Invitrogen). Nuclei were counterstained with DAPI. Images were obtained using the Leica WLL laser scanning confocal microscope.

### Subcutaneous tumor studies

All animal experimental procedures (SIBCB-S323-2202-04) were reviewed and approved by the Institutional Animal Care and Use Committee (IACUC) of the Center for Excellence in Molecular Cell Science (CEMCS), CAS. Female BALB/c nude mice, 6 weeks old and with weight ranging from 18 to 22 g, were supplied by Shanghai Jihui Laboratory Animal Care Co., Ltd. The mice were maintained in a pathogen-free environment (23 ± 2 °C and 55 ± 5% humidity) on a 12 h light-12 h dark cycle with food and water supplied ad libitum throughout the experimental period. The mice were randomly assigned to indicated groups. No statistical methods were employed to estimate sample size. Mice were subcutaneously inoculated with 2 × 10^6^ WT and modified A549 cells in a volume of 100 µl in the hind flanks. For ICAM-1‒neutralizing antibody treatment, anti‒ICAM-1 (JH12) or human IgG isotype control antibody was intraperitoneally administered at 150 μg per injection every two days until the end of the experiment. No animals were excluded. The longest diameter (a) and shortest diameter (b) of tumors were measured using digital calipers every 3 days, and tumor volume (V = 0.5 × a × b^2^) was calculated. The investigators were not blinded during experiments and outcome assessment.

### Terminal deoxynucleotidyl transferase (TdT)-mediated dUTP nick end labeling (TUNEL) assay

Freshly isolated subcutaneous xenografts were fixed in 4% PFA, embedded in OCT, sectioned at 8 μm thickness, and stained for apoptotic cells using a One Step TUNEL Apoptosis Assay Kit according to the manufacturer’s instructions. Sections were counterstained with DAPI (1:10000) for nuclei. The slides were photographed with a confocal laser scanning microscope (TCS SP8 WLL, Leica).

### Single-chain fragment variable (scFv) antibody screening

Tomlinson I and J Human Single-Fold scFv libraries were used for antibody screening against ICAM-1 Lys8-Ser22 peptide (KVILPRGGSVLVTCS) according to the manufacturer’s instruction. Phage clones with specific peptide-binding affinity were obtained after three rounds of panning. The binding of candidate scFv to rhICAM-1 protein was confirmed by ELISA. The VH and VL sequences were amplified from candidate scFv using vector-specific primers (LMB3: 5′-CAGGAAACAGCTATGAC-3′; pHENseq: 5′-CTATGCGGCCCCATTCA-3′) and were then cloned into plasmids containing the constant regions of human IgG1 heavy chain and Igκ light chain respectively to generate full length IgG1 mAb constructs.

### Bio-layer Interferometry (BLI) assay

To detect the binding affinity of JH12 IgG to ICAM-1 protein, BLI experiment was performed using an Octet Red 96 instrument (ForteBio, Fremont, USA). Briefly, biotinylated rhICAM-1 D1D2 (30 ng/μl) was immobilized on streptavidin (SA) biosensors and then incubated with gradient concentrations of JH12 IgG (500 nM to 6.25 nM) in PBS. The association and dissociation steps were both set to 300 s. The K_D_ value of ICAM-1 D1D2 binding affinity for JH12 IgG was calculated from the binding curves based on the global fit to a 1:1 Langmuir binding model with an R2 value of ≥ 0.95. The kinetically derived affinities were calculated as K_D_ = k_off_/k_on_. Binding experiments were performed at 25 °C. Data were analyzed using Octet Data Analysis Software 9.0 (ForteBio, Menlo park, CA, USA).

### Soluble ligand binding assay

FG protein was labeled with Alexa Fluor 488 dye using Protein Labeling Kit (A10235, Invitrogen). A549 cells were pre-incubated with serial concentrations of JH12 or human IgG control for 30 min at 4 °C, 100 nM Alexa Fluor 488-labelled FG protein was then added to the mixture for 30 min at room temperature.

For inhibition of soluble ICAM-1 binding to β2 integrins by JH12 mAb, rhICAM-1–Flag (50 μg/ml) was labeled with APC-conjugated anti-Flag tag antibody and followed by treatment with the indicated concentrations of JH12 or 25 μg/ml human IgG control in HBS buffer containing 1 mM Ca^2+^ + Mg^2+^ for 30 min at 4 °C before incubation with NK-92 cells for 30 min at room temperature. Cells were washed twice before flow cytometry analysis. The results were analyzed by FlowJo and IC_50_ was determined by non-linear regression analysis using GraphPad Prism.

### Statistical analysis

For each experiment, sample size was chosen on the basis of similar experimental approaches reported in the literature. All data were tested using the Shapiro-Wilk and Kolmogorov-Smirnov normality tests. For Gaussian data, pairwise comparisons were performed using unpaired Student’s *t*-test or Welch’s unequal variance *t*-test after variance homogeneity tests using the F test. Comparisons between three or more groups were performed using ordinary one-way ANOVA or Brown-Forsythe and Welch one-way ANOVA followed by Dunnett’s test for multiple comparisons after variance homogeneity tests using the Brown-Forsythe test. Group analysis with two variables was carried out using two-way ANOVA. All statistical analysis was calculated using GraphPad Prism 9 (GraphPad Software). The resulting P values are indicated as follows: NS, *P* > 0.05; *, 0.01 < *P* < 0.05; **, 0.001 < *P* < 0.01; ***, *P* < 0.001.

### Supplementary information

Supplementary Figure Legends

Supplementary Figure

original western blots

## Data Availability

The data that support the findings of this study are available from the corresponding author upon reasonable request.
