# Supplementary material for: Neoplastic ICAM-1 protects lung carcinoma from apoptosis through ligation of fibrinogen
Source: Cell Death Dis. 2024 Aug 21;15(8):605. doi: 10.1038/s41419-024-06989-9 (PMC11339363; doi:10.1038/s41419-024-06989-9)
Supplement: Supplementary file 1 — Supplementary Figure Legends [file 41419_2024_6989_MOESM1_ESM.docx]

**Supplementary Figure Legends**

**Supplementary Fig. S1 Tumor cells in human NSCLC show dominant ICAM-1 expression.** ICAM-1 expression within human NSCLC was obtained through bioinformatics analysis of publicly available core NSCLC atlas including malignant, epithelial, immune and stromal/endothelial components (https://cellxgene.cziscience.com/collections/edb893ee-4066-4128-9aec-5eb2b03f8287). A, Uniform manifold approximation and projection (UMAP) of 360,038 cells from scRNA-seq of 159 human NSCLC samples across 8 datasets. B, UMAP showing expression of ICAM-1 within different cell populations.

**Supplementary Fig. S2 Cell surface expression of ICAM-1 was determined by flow cytometry in 16HBE, A549 and H1650 cells.**

Quantification of ICAM-1 expression as a ratio relative to the expression level of 16HBE is shown (right panel). Data are represented as mean ± SD, n = 3. One-way ANOVA with Dunnett’s multiple comparisons test. All data are from three independent experiments. ***, P < 0.001.

**Supplementary Fig. S3 ICAM-1 knockdown in 16HBE, A549 and H1650 cells.**

16HBE, A549 and H1650 cells were stably transfected with ICAM-1 shRNAs. ICAM-1 surface expression was examined by flow cytometry. Quantification of ICAM-1 expression is shown (lower panel). Data are represented as mean ± SD, n = 3. One-way ANOVA with Dunnett’s multiple comparisons test. All data are from three independent experiments. *, P < 0.05; **, P < 0.01; ***, P < 0.001.

**Supplementary Fig. S4 ICAM-1 overexpression (O/E) does not affect A549 and H1650 cell apoptosis.**

A, A549 and H1650 cells were transfected with ICAM-1. ICAM-1 surface expression was examined by flow cytometry. Quantification of ICAM-1 expression is shown (right panel). Data are represented as mean ± SD, n = 3. Unpaired two-tailed Welch’s unequal variance *t*-test. B, Flow cytometric analyses of apoptotic cells with ICAM-1 overexpression (O/E). A representative result of three independent experiments is shown. Living cells tested negative for both Annexin V-FITC and PI. Populations testing FITC^+^/PI^-^ were classified as early-stage apoptotic cells, and double-positive cells were classified as late-stage apoptotic cells. Right panel, bar graphs quantifying the percentage of early-stage apoptotic and late-stage apoptotic cells. Data are represented as mean ± SD, n = 3. Unpaired two-tailed Student’s *t*-test. All data are from three independent experiments. ***, P < 0.001; NS, P > 0.05.

**Supplementary Fig. S5 ICAM-1 and FGG associates with poor prognosis in some cancer types.**

A, NSCLC patients with high FGG expression present a worse progression-free survival (P < 0.001) and overall survival (P < 0.01) compared with patients with low FGG expression from GEO, EGA and TCGA databases. B, Kaplan-Meier survival analysis of cohorts of colorectal, [pancreatic](javascript:;) and renal cancer patients from GEO, EGA and TCGA databases shows that high ICAM-1 (upper panel) or FGG (lower panel) expression is correlated with worse overall survival (https://kmplot.com/analysis/).

**Supplementary Fig. S6 Neoplastic FGG correlates with ICAM-1 expression in NSCLC.**

A, Spearman’s correlation analysis of ICAM-1 and FGG expression levels in TCGA NSCLC patient cohort (http://gepia.cancer-pku.cn/). r and p-values were calculated using Spearman’s rank correlation test. B, FGG staining patterns in NSCLC specimens from the Human Protein Atlas (HPA) (http://www.proteinatlas.org/). Upper panel, representative images of FGG positive (medium- and low-expression) NSCLC specimens. Scale bar, 200 µm. Lower panel, staining and FGG expression cell type plots from the HPA.

**Supplementary Fig. S7 ICAM-1 and FGG expression in A549 xenografts.**

Expression levels of ICAM-1 and FGG were determined by immunoblotting in A549 xenografts from the indicated groups. A representative result of three independent experiments is shown.

**Supplementary Fig. S8 JH12 mAb does not affect ICAM-1 binding to integrin αLβ2 or αMβ2.**

A, Cell surface expression of integrin αL, αM and β2 on NK-92 cells was measured by flow cytometry. The numbers within the figure show the specific mean fluorescence intensities of HI111 (anti-αL) mAb, M1/70 (anti-αM) mAb and TS1/18 (anti-β2) mAb. A representative result of three independent experiments is shown. B, Binding of soluble ICAM-1 to NK-92 cells in the presence of antibody JH12 or human IgG control. Data are represented as mean ± SD, n = 3. One-way ANOVA with Dunnett’s multiple comparisons test. All data are from three independent experiments. NS, P > 0.05.

**Supplementary Fig. S9 Combined JH12 mAb and ERK1/2 inhibitor SCH772984 presents a synergistic pro-apoptosis effect.**

A, Apoptosis of A549 cells treated with human IgG control, JH12, DMSO vehicle control, SCH772984 and combined JH12 plus SCH772984 was examined by Annexin V-FITC/PI staining assay. Dot plots for flow cytometric analyses of apoptotic cells are shown in the left panel. A representative result of three independent experiments is shown. Data are represented as mean ± SD, n = 3. One-way ANOVA with Dunnett’s multiple comparisons test. All data are from three independent experiments. B, Immunoblotting of caspase-9/3 in A549 cells treated human IgG control, JH12, DMSO vehicle control, SCH772984 and combined JH12 plus SCH772984. A representative result of three independent experiments is shown. ***, P < 0.001; NS, P > 0.05.

**Supplementary Fig. S10 Knockdown of ICAM-1 significantly induces apoptosis of colorectal, pancreatic and renal cancer cells.**

A, Colon adenocarcinoma cell lines SW480, HT-29, HCT 116, pancreatic adenocarcinoma cell line PANC-1 and renal cell carcinoma cell lines 769-P and 786-O were transfected with control scrambled or ICAM-1–targeted shRNAs. Cell surface expression of ICAM-1 was examined by flow cytometry. Quantification of three independent experiments for ICAM-1 expression is shown. B, Left panel, dot plots for flow cytometric analyses of apoptotic cells. A representative result of three independent experiments is shown. Right panel, bar graphs quantifying the percentage of FITC^+^/PI^-^ early-stage apoptotic and FITC^+^/PI^+^ late-stage apoptotic cells. Data are represented as mean ± SD, n = 3. Unpaired two-tailed Student’s *t*-test and unpaired two-tailed Welch’s unequal variance *t*-test. All data are from three independent experiments. **, P < 0.01; ***, P < 0.001.
