# Supplementary material for: Neoplastic ICAM-1 protects lung carcinoma from apoptosis through ligation of fibrinogen
Source: Cell Death Dis. 2024 Aug 21;15(8):605. doi: 10.1038/s41419-024-06989-9 (PMC11339363; doi:10.1038/s41419-024-06989-9)
Supplement: Supplementary file 2 — Supplementary Figure [file 41419_2024_6989_MOESM2_ESM.pdf]

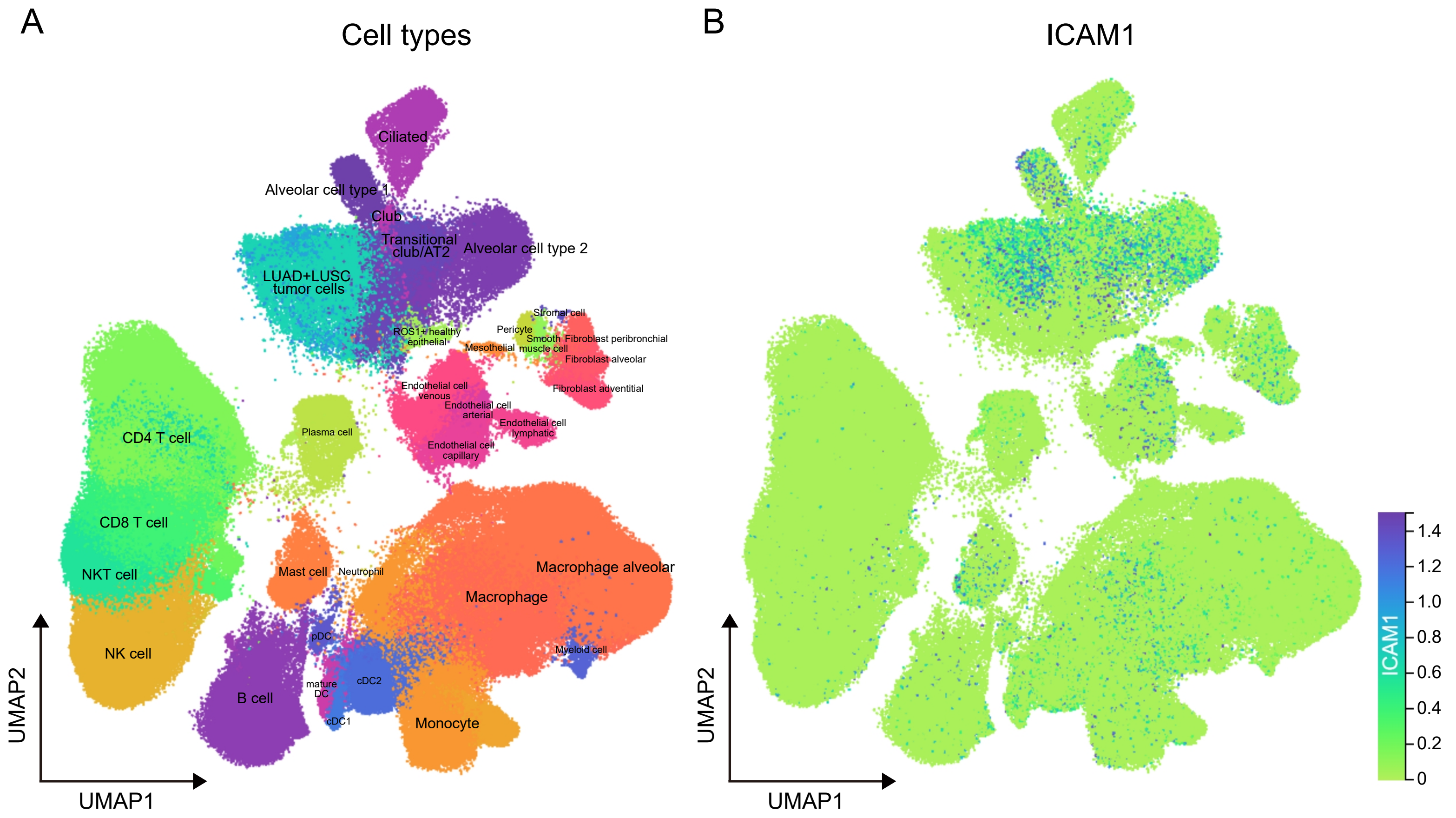

Supplementary Fig. S1

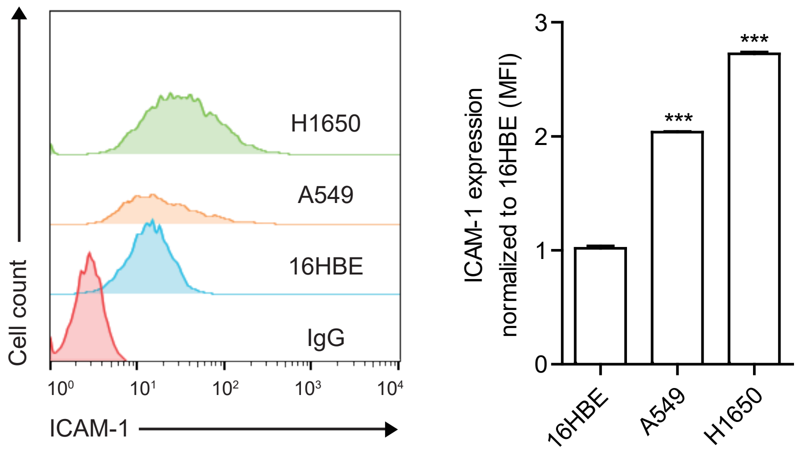

Supplementary Fig. S2

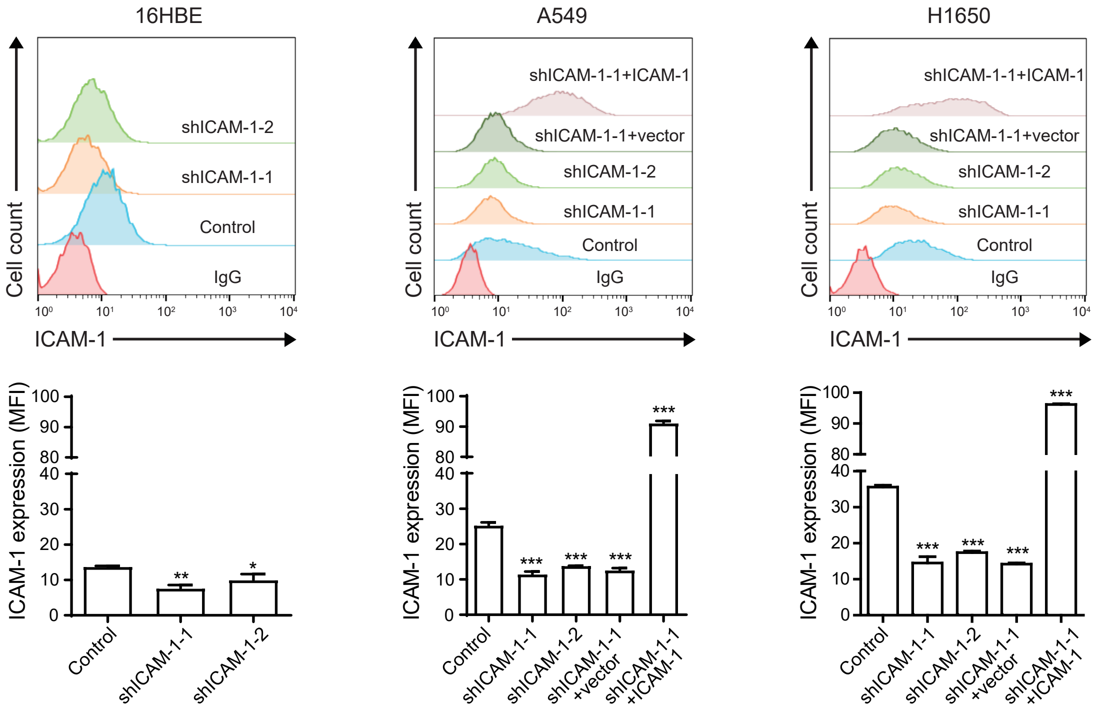

Supplementary Fig. S3

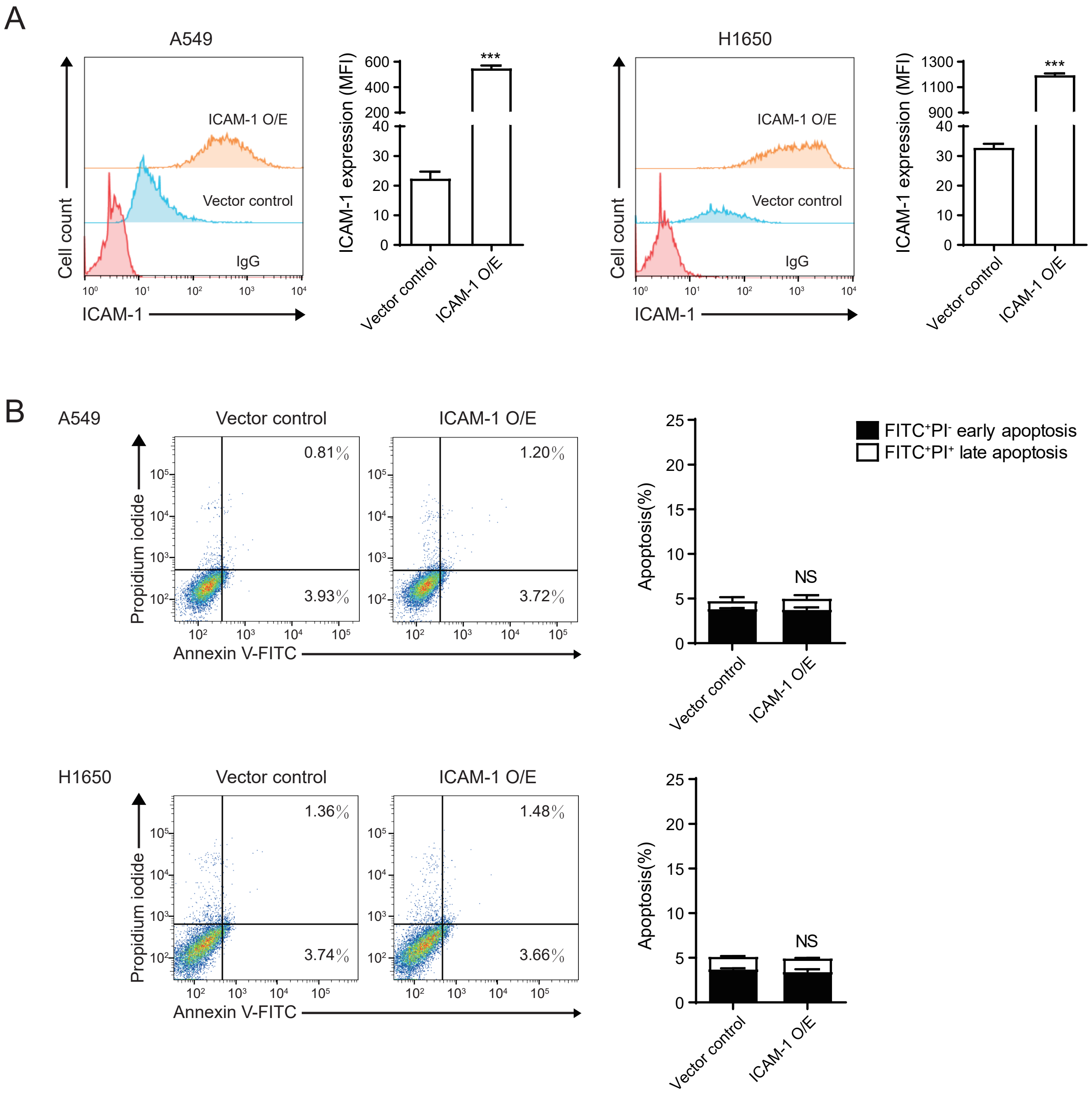

Supplementary Fig. S4

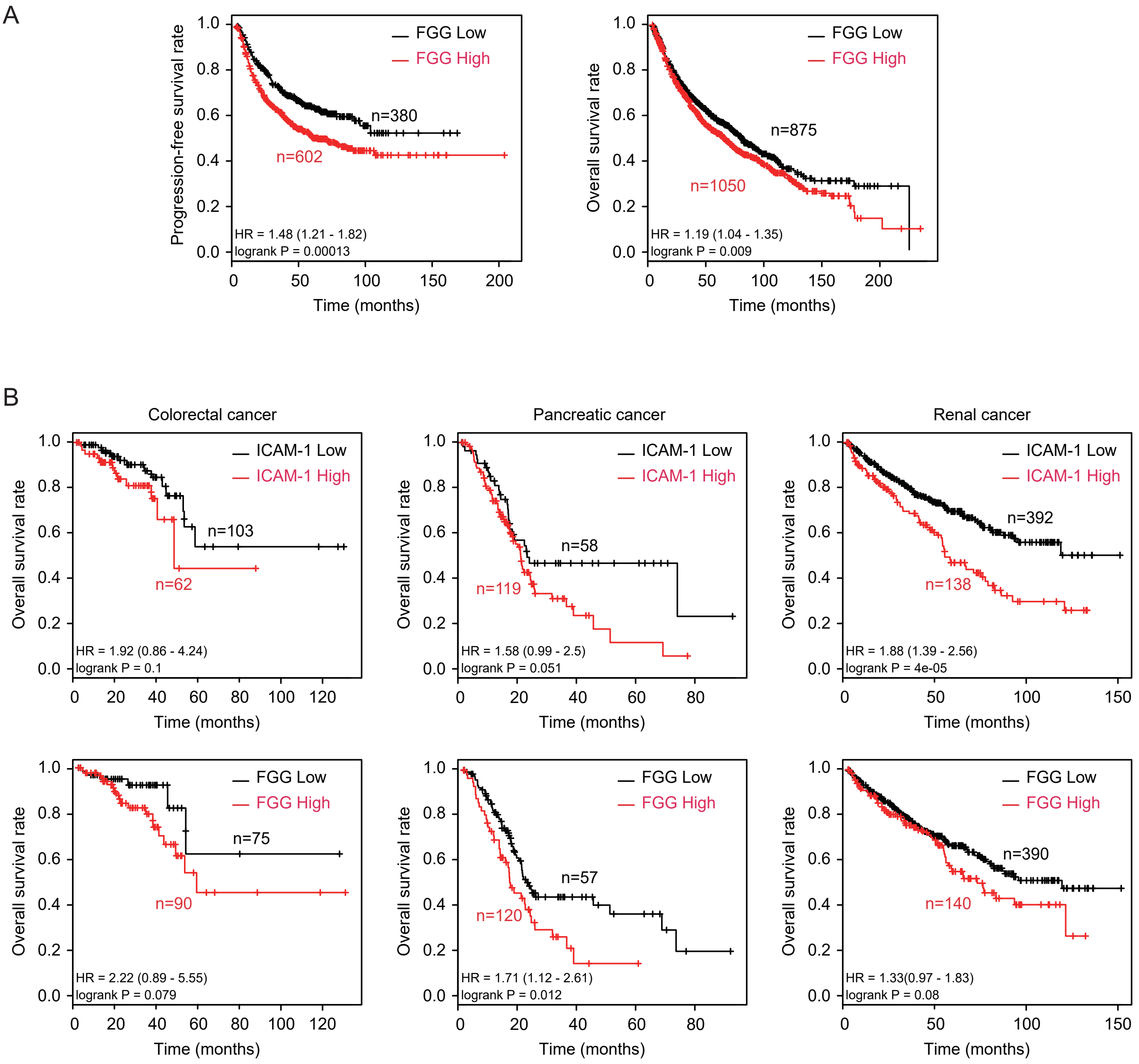

Supplementary Fig. S5

A

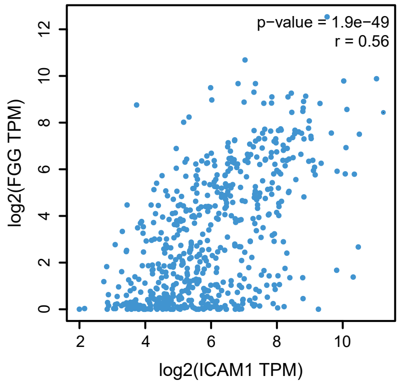

B

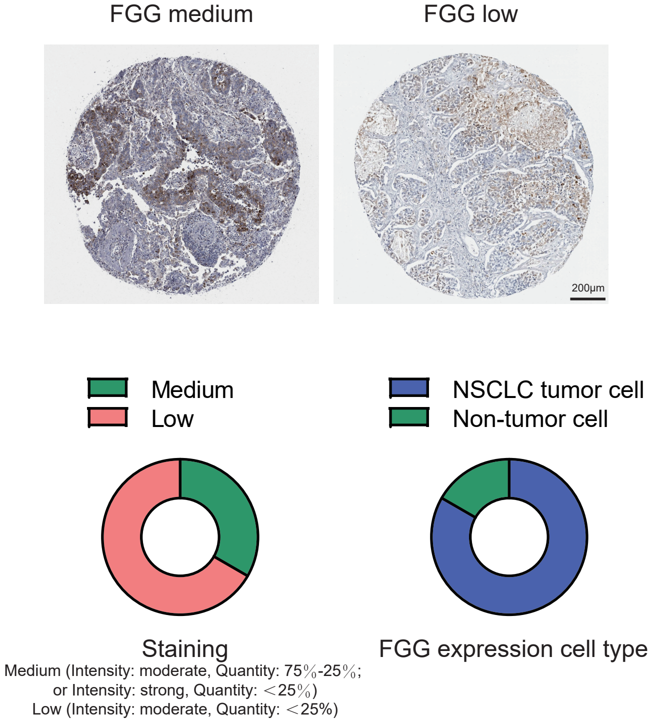

Supplementary Fig. S6

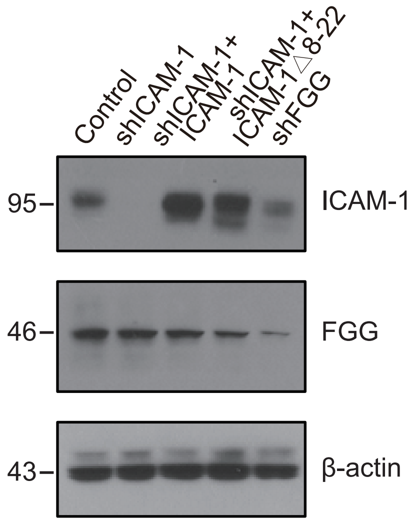

Supplementary Fig. S7

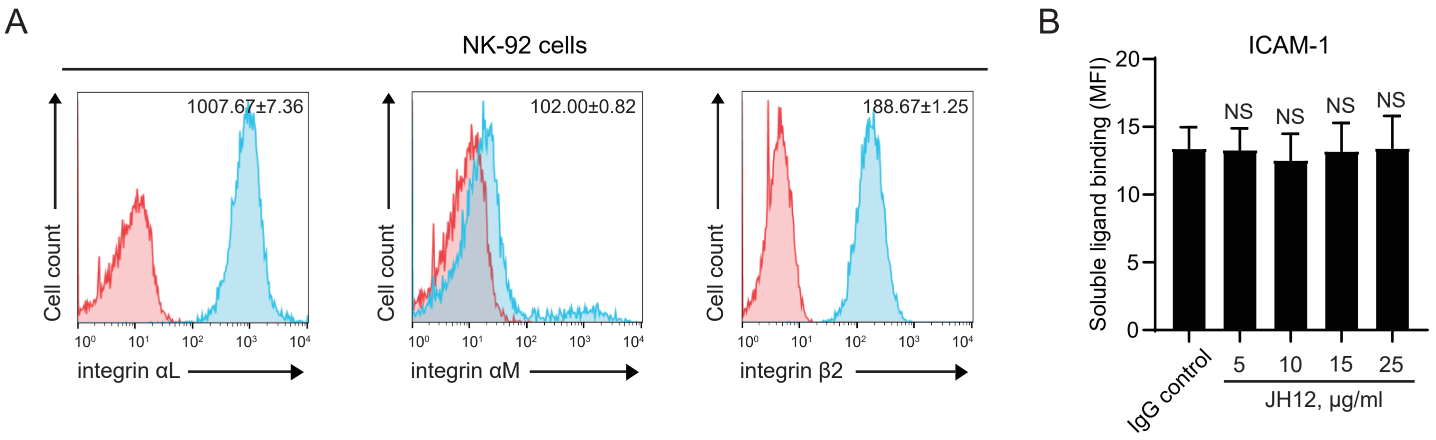

Supplementary Fig. S8

A

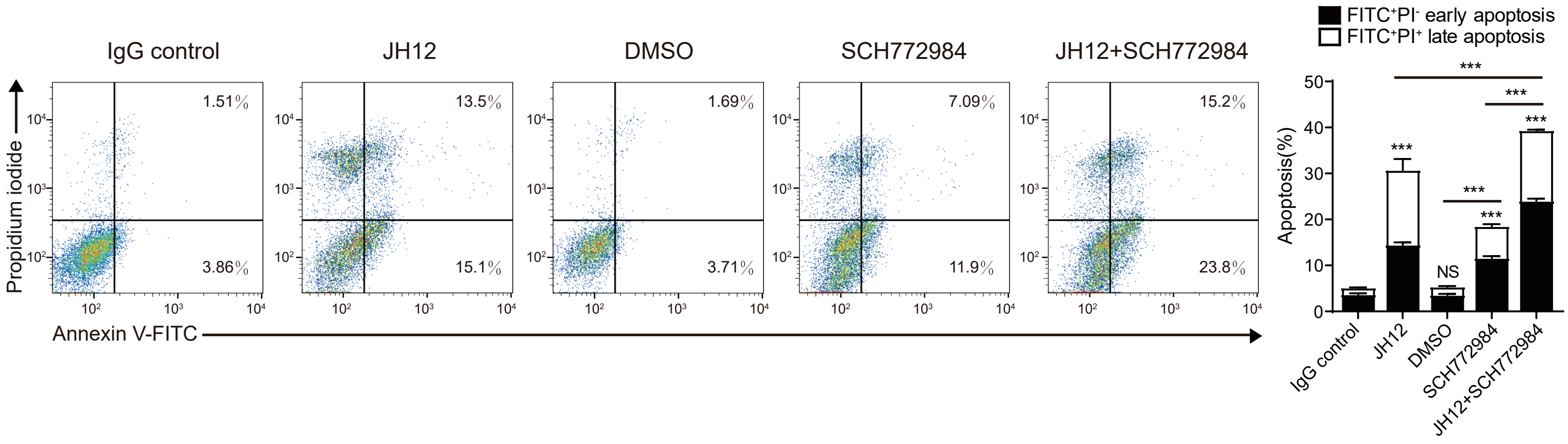

B

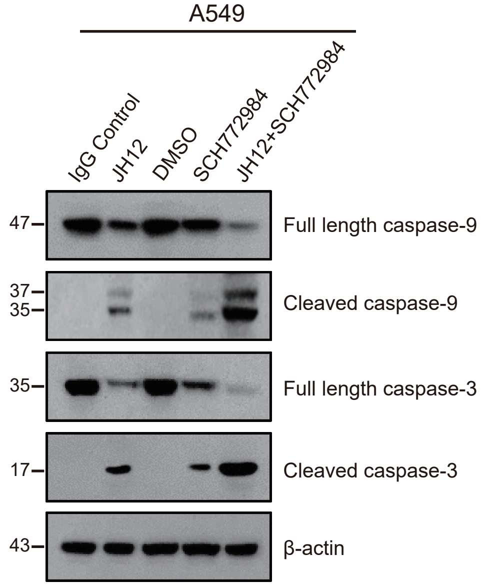

Supplementary Fig. S9

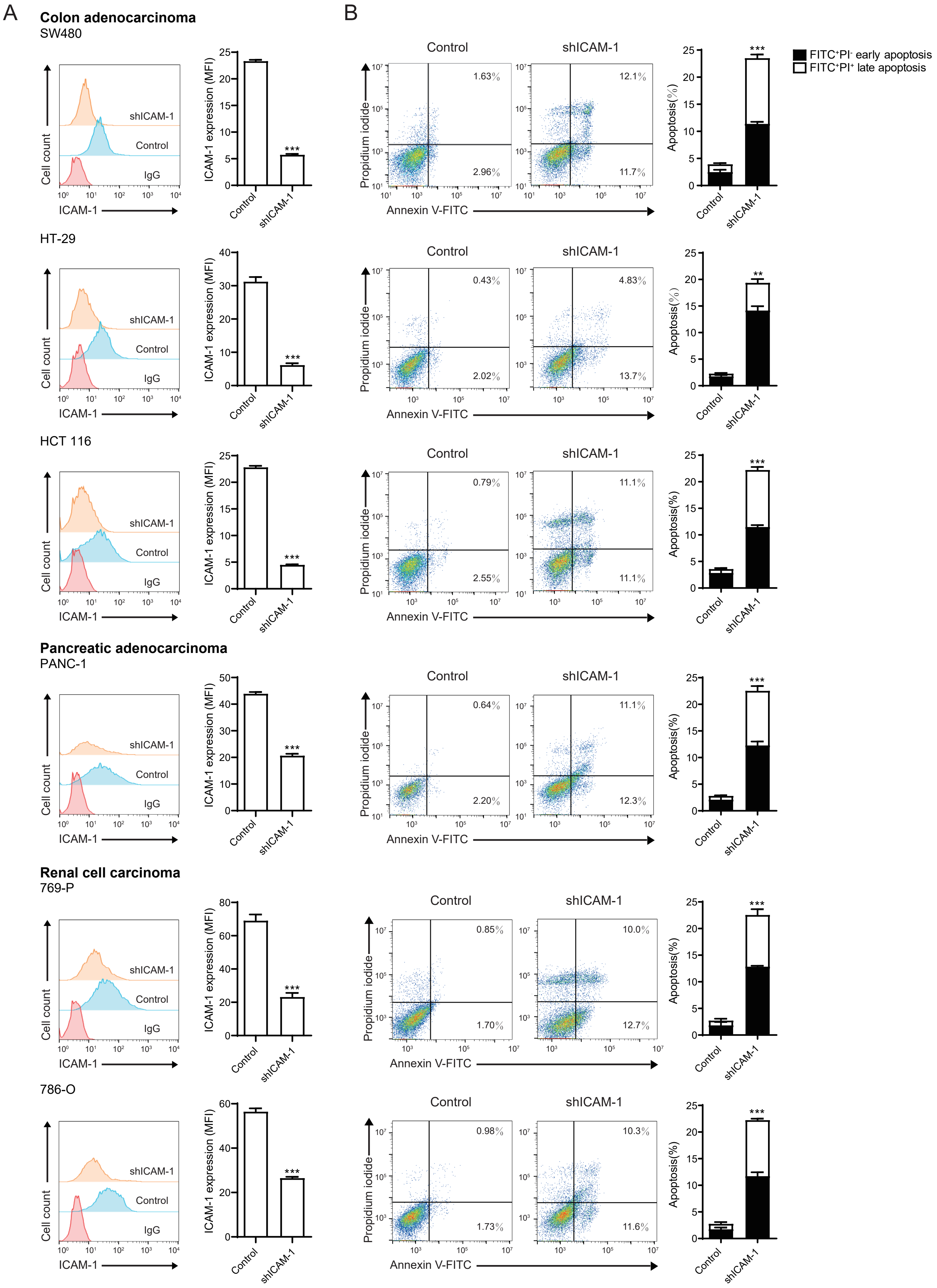

Supplementary Fig. S10
