# Supplementary material for: Neoplastic ICAM-1 protects lung carcinoma from apoptosis through ligation of fibrinogen
Source: Cell Death Dis. 2024 Aug 21;15(8):605. doi: 10.1038/s41419-024-06989-9 (PMC11339363; doi:10.1038/s41419-024-06989-9)
Supplement: Supplementary file 3 — original western blots [file 41419_2024_6989_MOESM3_ESM.pdf]

Figure 3B

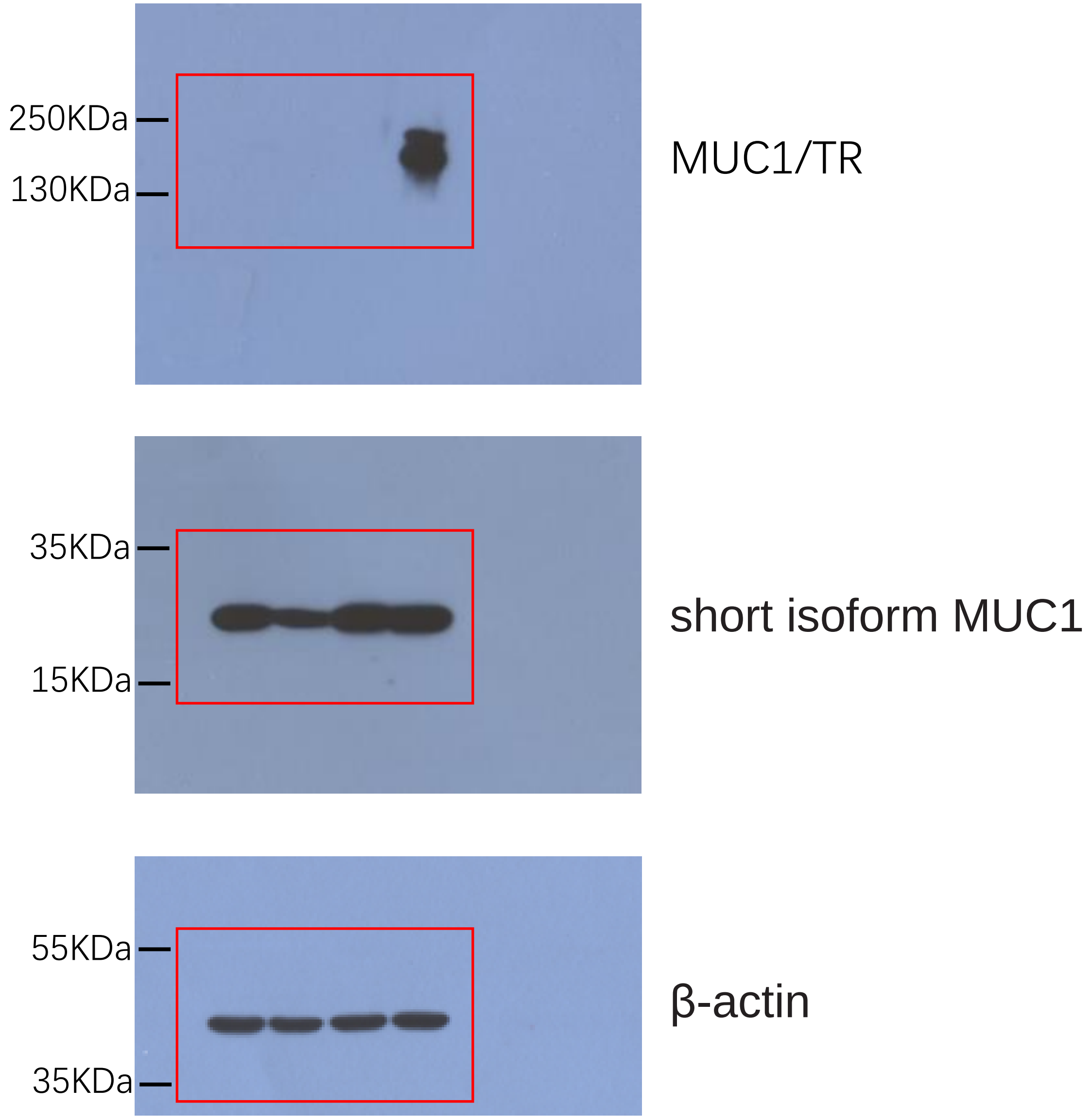
250KDa
130KDa
MUC1/TR
35KDa
15KDa
short isoform MUC1
55KDa
35KDa
β-actin

Figure 3C

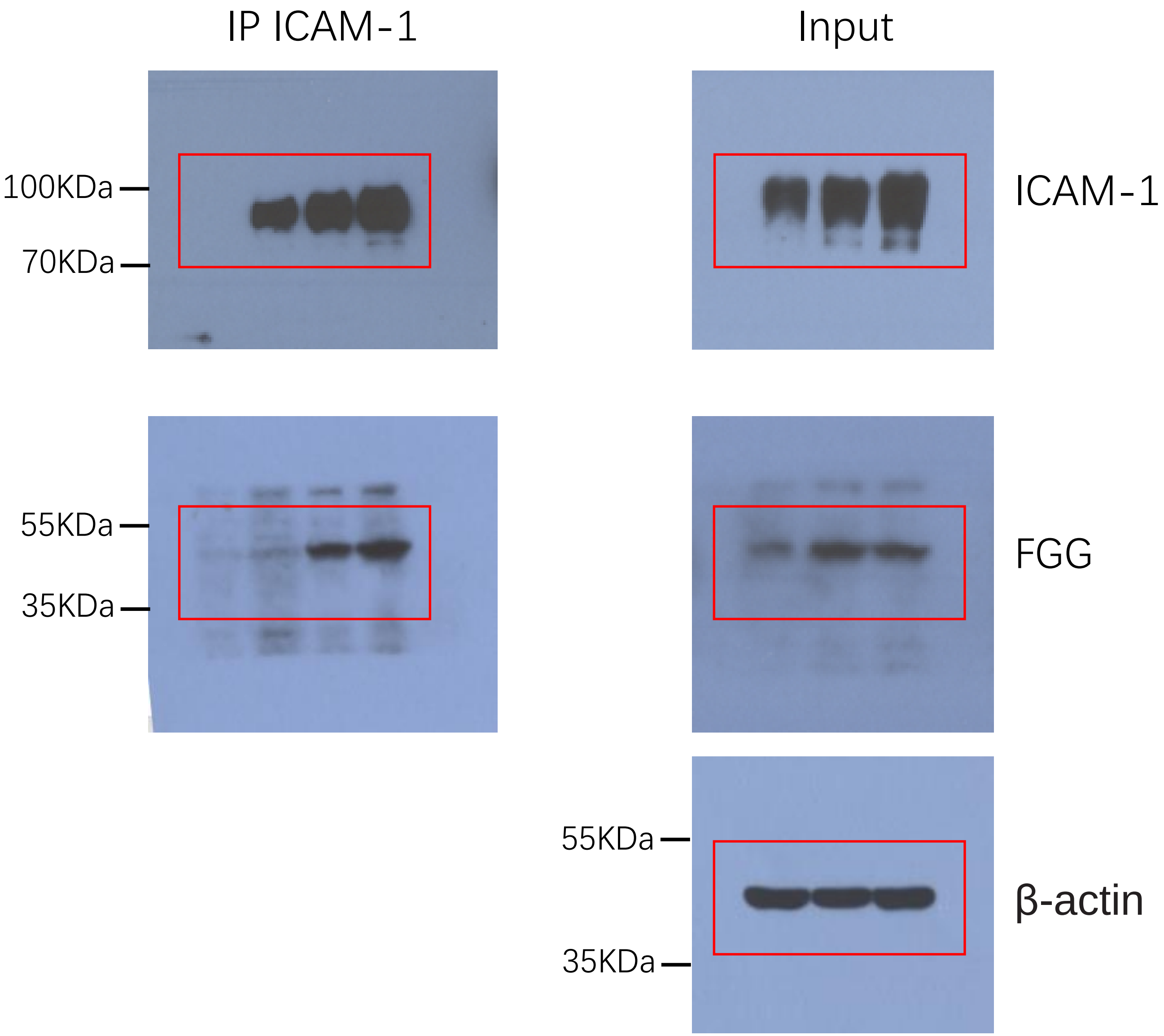

IP ICAM-1
Input
100KDa
70KDa
ICAM-1
55KDa
35KDa
FGG
55KDa
35KDa
β-actin

Figure 3E

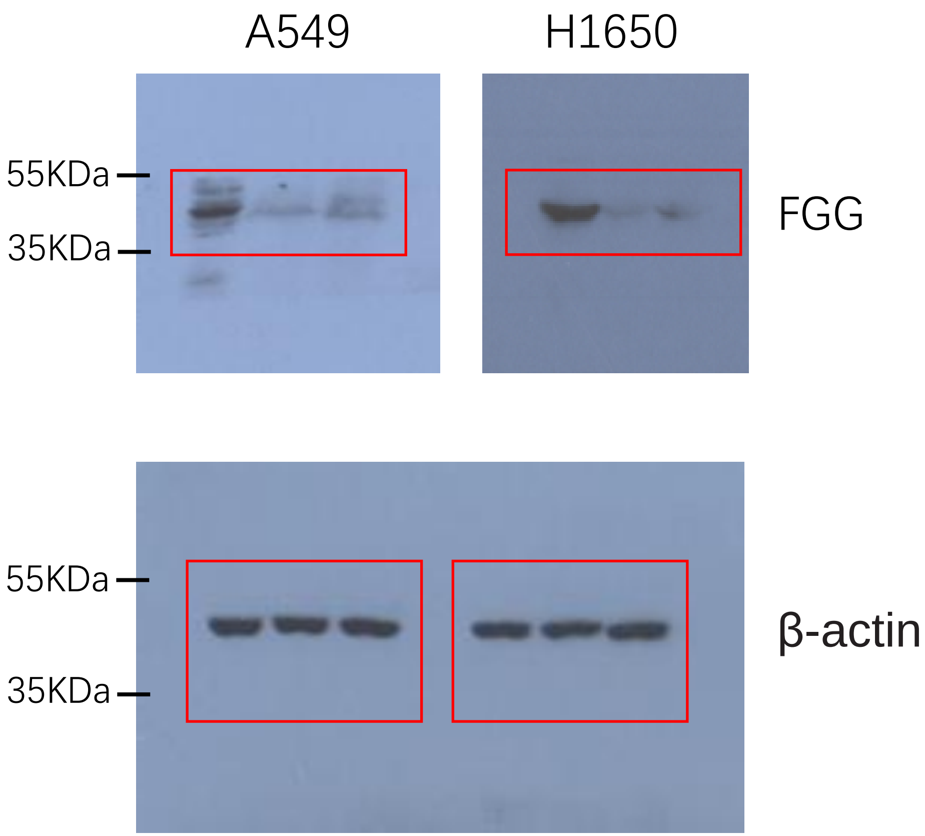

Figure 5A

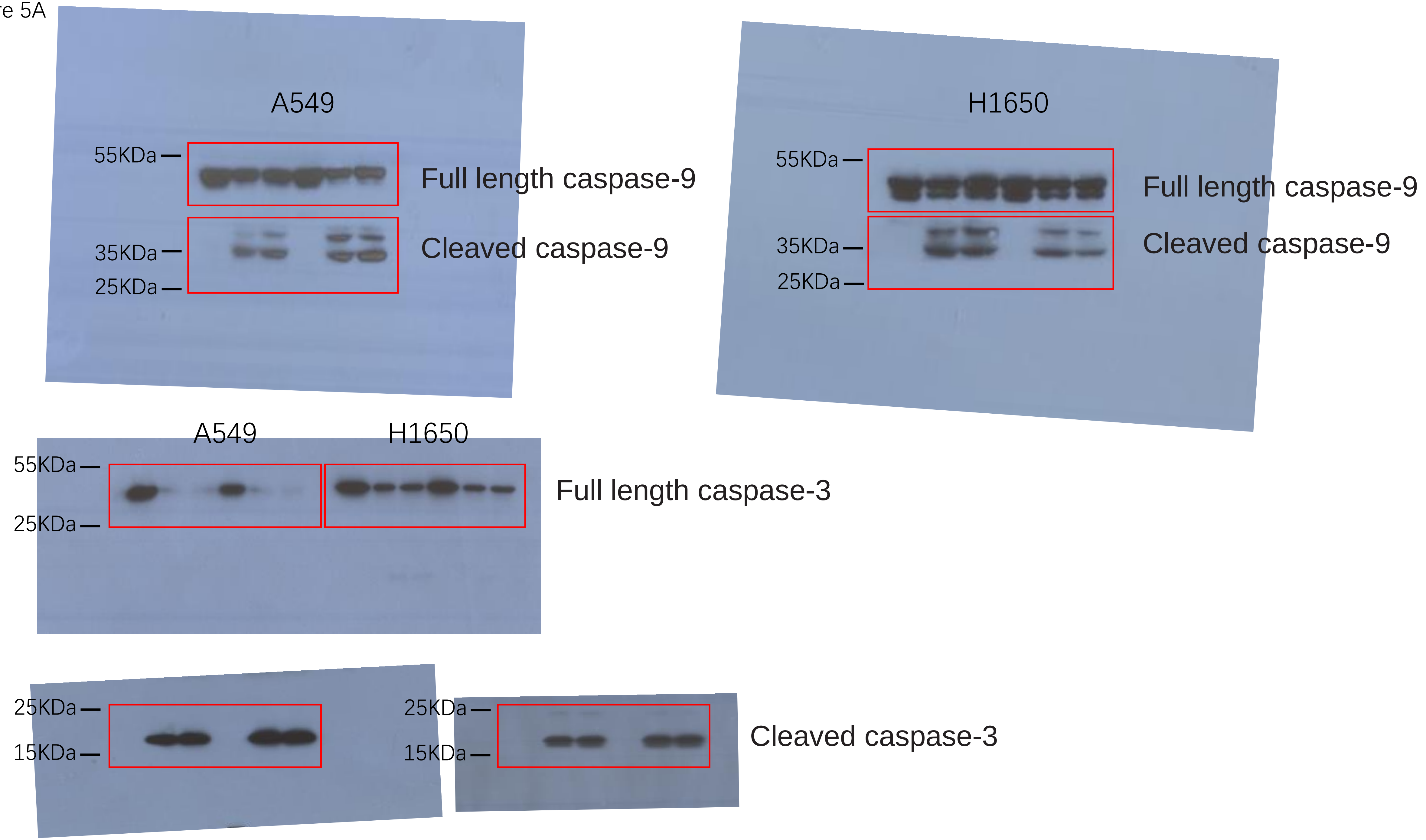

A549
55KDa
35KDa
25KDa
Full length caspase-9
Cleaved caspase-9
H1650
55KDa
35KDa
25KDa
Full length caspase-9
Cleaved caspase-9
A549
H1650
55KDa
25KDa
Full length caspase-3
25KDa
15KDa
25KDa
15KDa
Cleaved caspase-3

Figure 5A

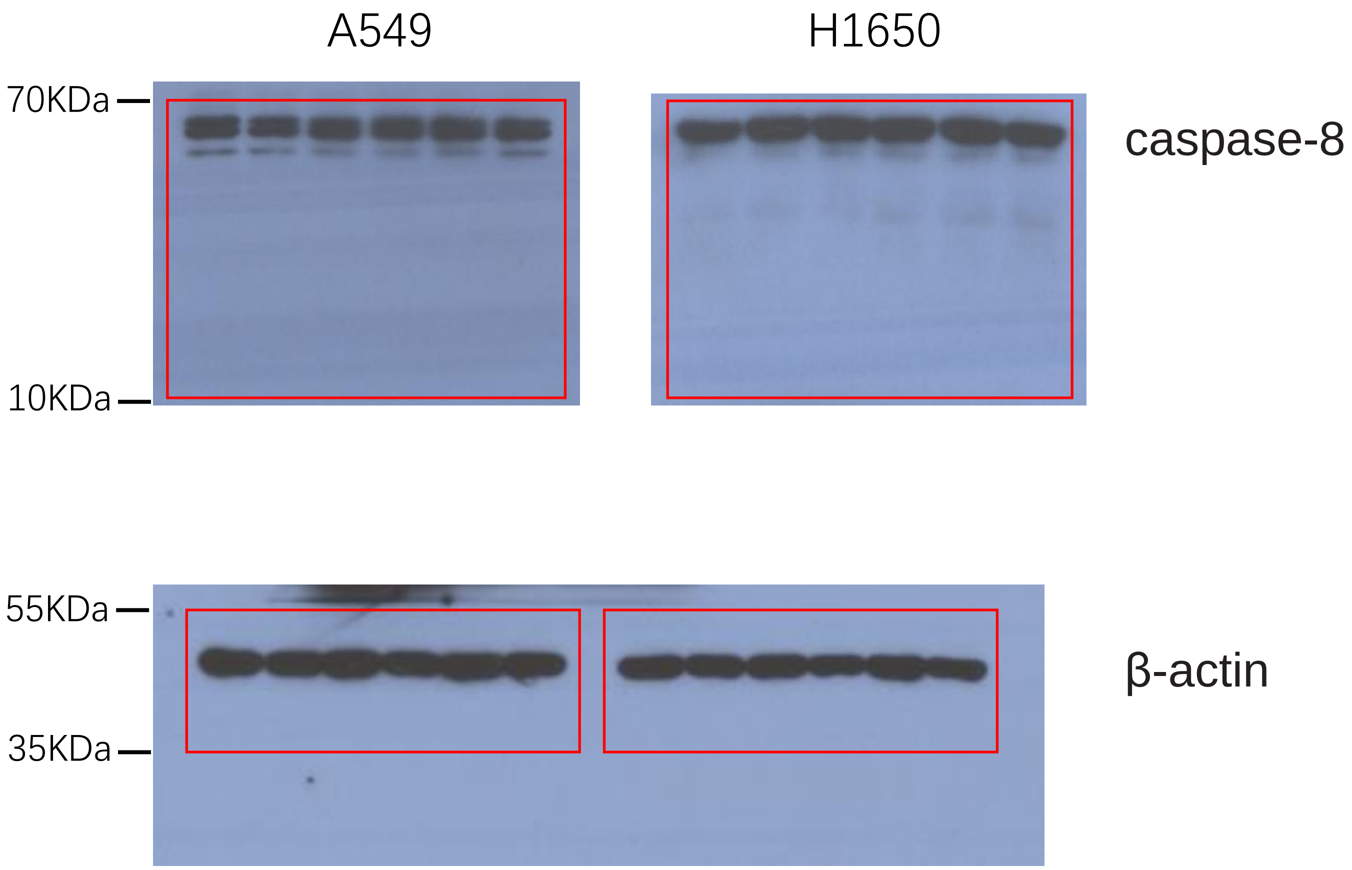

Figure 5B

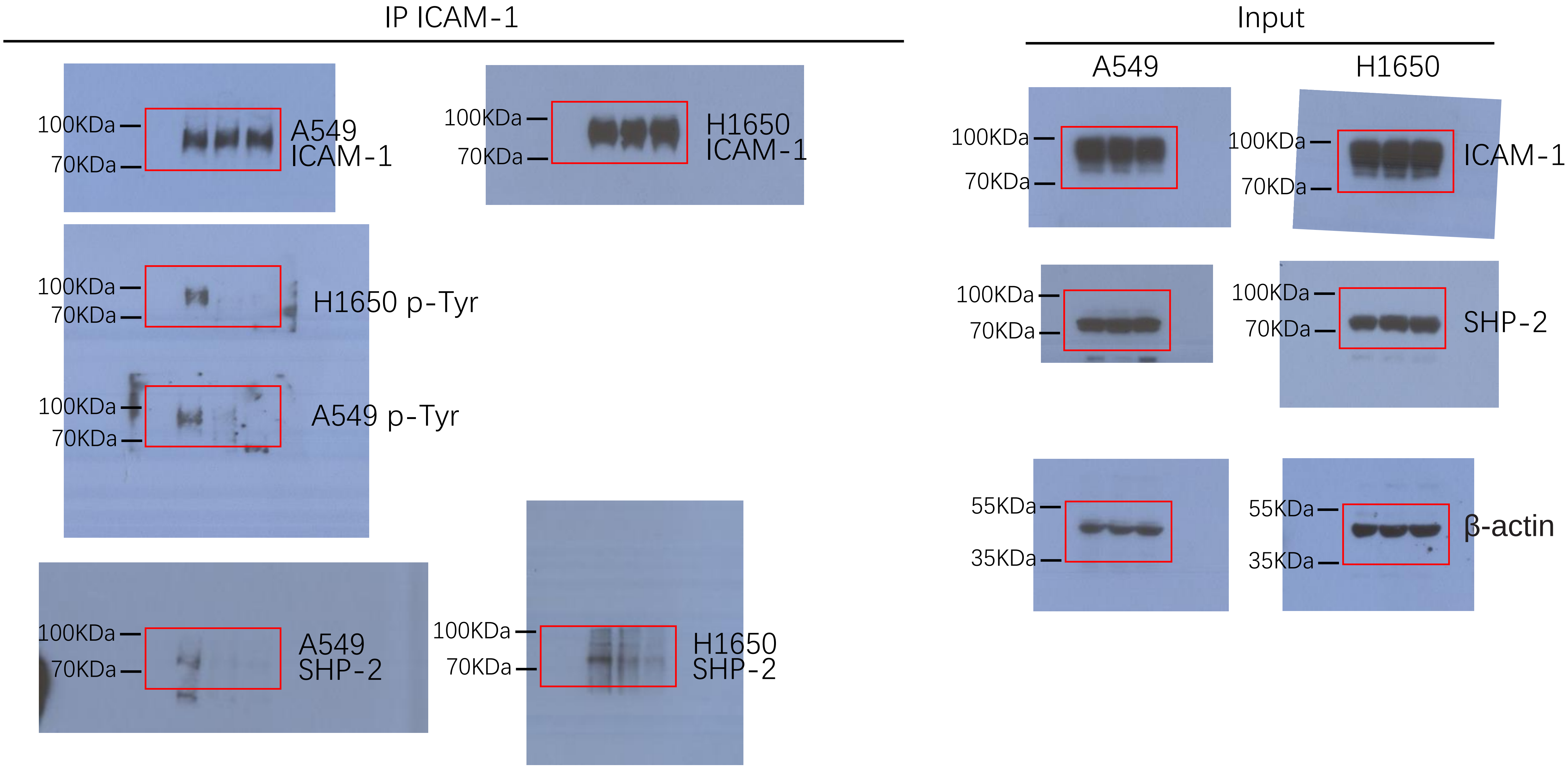
IP ICAM-1
Input
A549
H1650
100KDa
70KDa
A549
ICAM-1
H1650
ICAM-1
H1650 p-Tyr
A549 p-Tyr
A549
SHP-2
H1650
SHP-2
ICAM-1
SHP-2
55KDa
35KDa
β-actin

Figure 5C

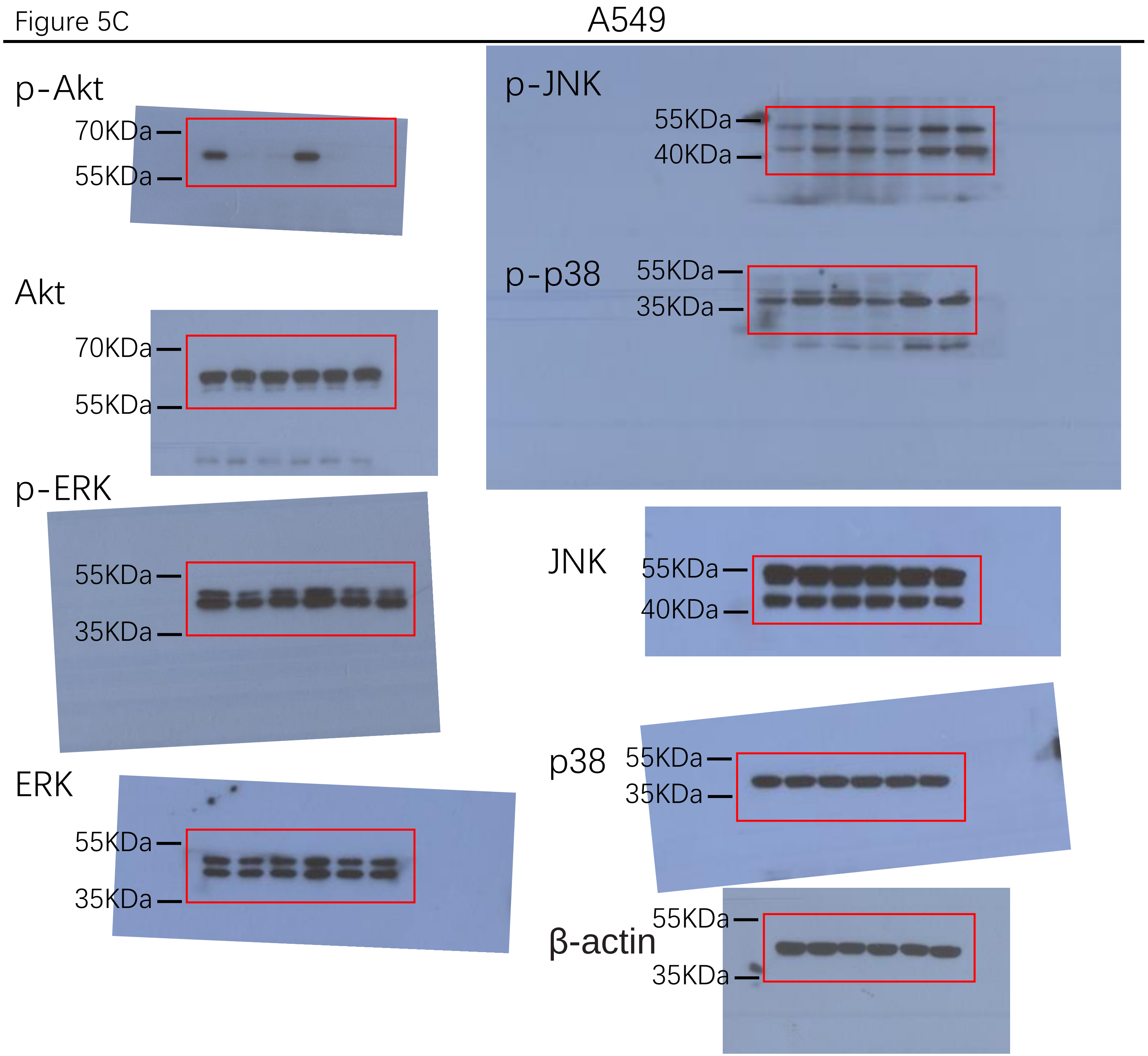
A549
p-Akt
70KDa
55KDa
Akt
70KDa
55KDa
p-ERK
55KDa
35KDa
ERK
55KDa
35KDa
p-JNK
55KDa
40KDa
p-p38
55KDa
35KDa
JNK
55KDa
40KDa
p38
55KDa
35KDa
β-actin
55KDa
35KDa

Figure 5C

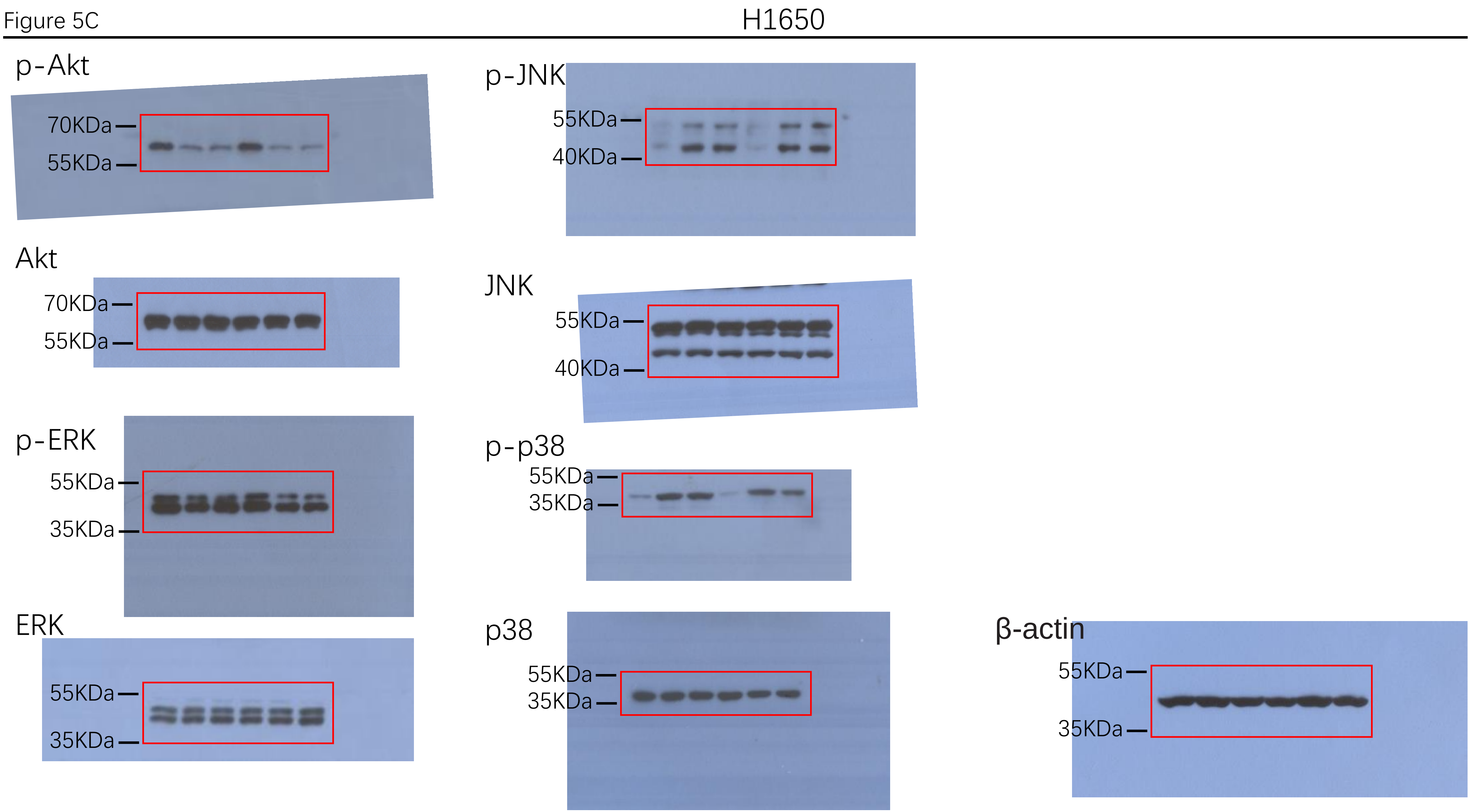
H1650
p-Akt
70KDa
55KDa
Akt
70KDa
55KDa
p-ERK
55KDa
35KDa
ERK
55KDa
35KDa
p-JNK
55KDa
40KDa
JNK
55KDa
40KDa
p-p38
55KDa
35KDa
p38
55KDa
35KDa
β-actin
55KDa
35KDa

Figure 6D

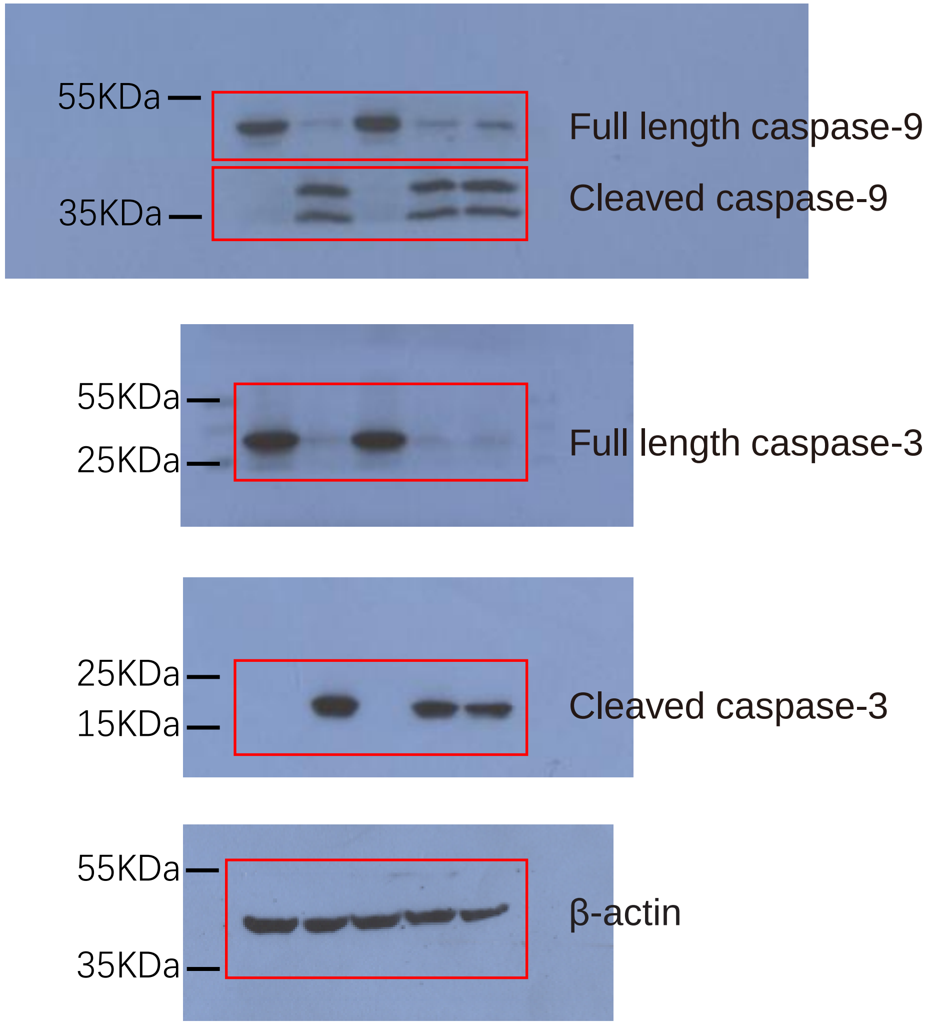

55KDa
35KDa
Full length caspase-9
Cleaved caspase-9
55KDa
25KDa
Full length caspase-3
25KDa
15KDa
Cleaved caspase-3
55KDa
35KDa
β-actin

Figure 7C

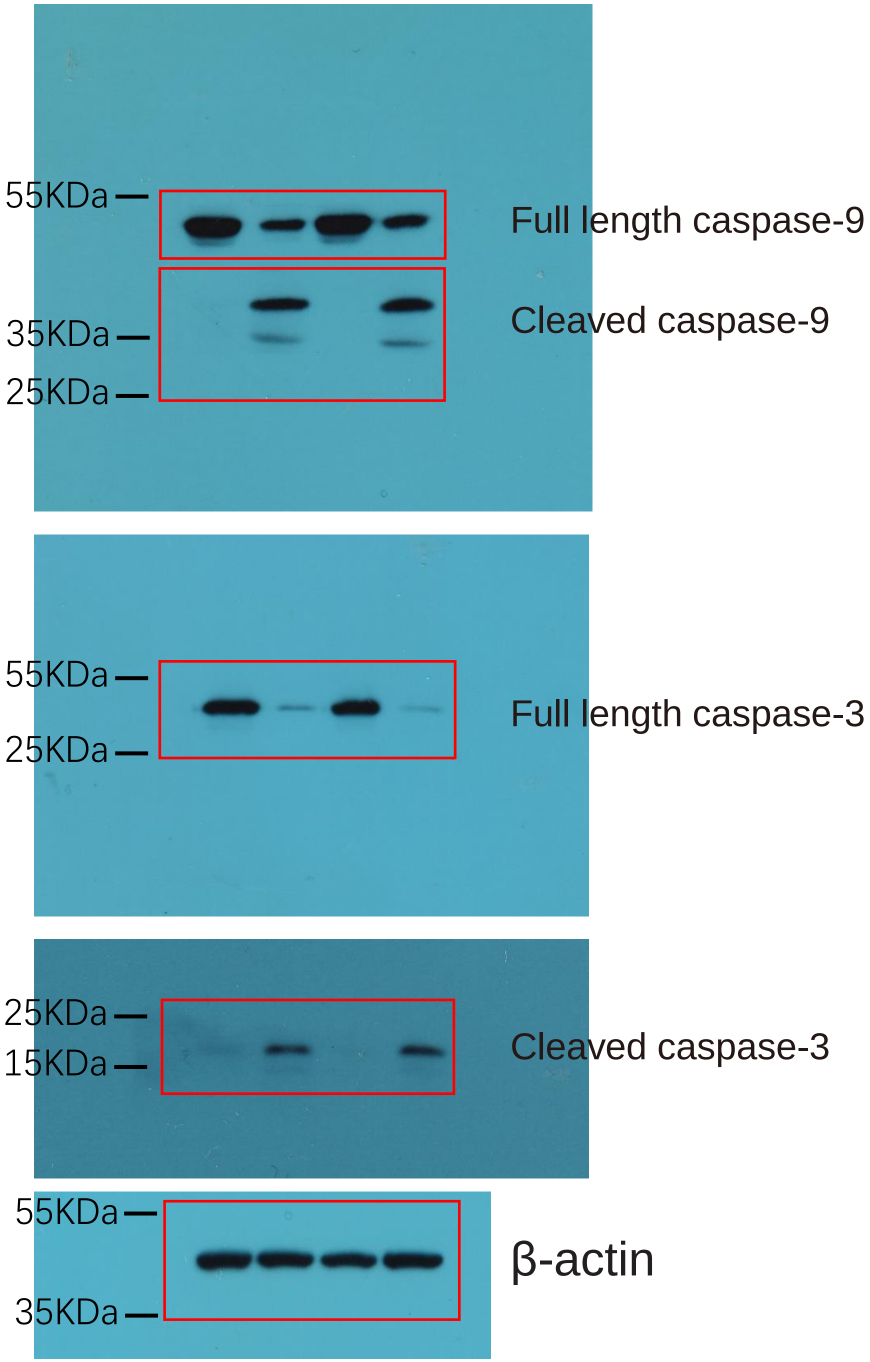

55KDa
35KDa
25KDa
Full length caspase-9
Cleaved caspase-9
55KDa
25KDa
Full length caspase-3
25KDa
15KDa
Cleaved caspase-3
55KDa
35KDa
β-actin

Figure S7

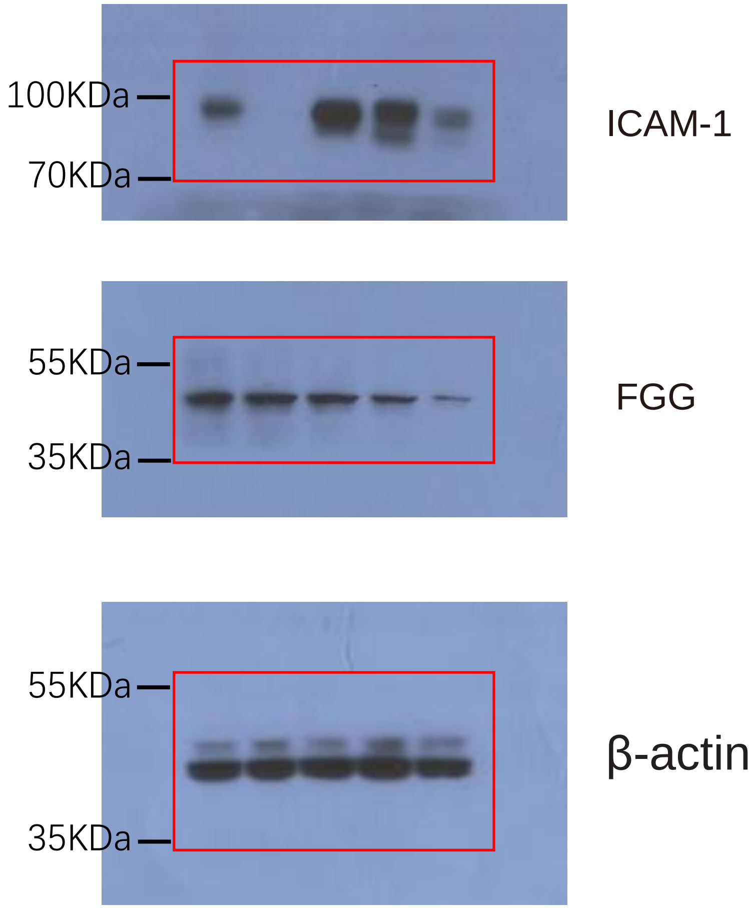

Figure S9B

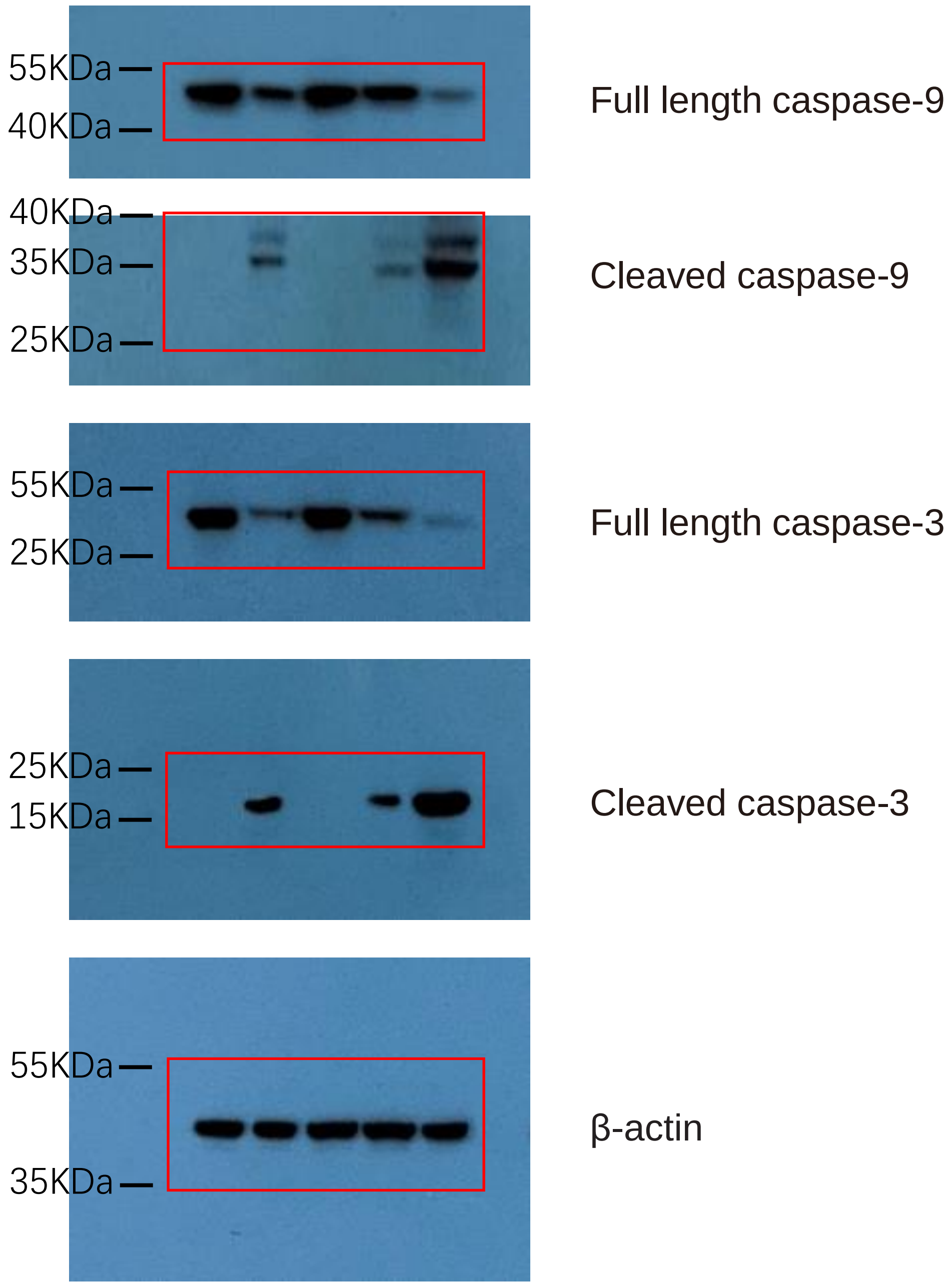
55KDa
40KDa
Full length caspase-9
40KDa
35KDa
25KDa
Cleaved caspase-9
55KDa
25KDa
Full length caspase-3
25KDa
15KDa
Cleaved caspase-3
55KDa
35KDa
β-actin
